# Prolonged breastfeeding protects from obesity by hypothalamic action of hepatic FGF21

**DOI:** 10.1038/s42255-022-00602-z

**Published:** 2022-07-25

**Authors:** Veronica Pena-Leon, Cintia Folgueira, Silvia Barja-Fernández, Raquel Pérez-Lois, Natália Da Silva Lima, Marion Martin, Violeta Heras, Sara Martinez-Martinez, Paola Valero, Cristina Iglesias, Mannon Duquenne, Omar Al-Massadi, Daniel Beiroa, Yara Souto, Miguel Fidalgo, Rasika Sowmyalakshmi, Diana Guallar, Juan Cunarro, Cecilia Castelao, Ana Senra, Patricia González-Saenz, Rocío Vázquez-Cobela, Rosaura Leis, Guadalupe Sabio, Helge Mueller-Fielitz, Markus Schwaninger, Miguel López, Sulay Tovar, Felipe F. Casanueva, Emmanuel Valjent, Carlos Diéguez, Vincent Prevot, Rubén Nogueiras, Luisa M. Seoane

**Affiliations:** 1 Endocrine Physiopathology Group, Instituto de Investigación Sanitaria de Santiago de Compostela, Complejo Hospitalario Universitario de Santiago/SERGAS, Santiago de Compostela, Spain; 2 CIMUS, University of Santiago de Compostela-Instituto de Investigación Sanitaria de Santiago de Compostela, Santiago de Compostela, Spain; 3 CIBEROBN Physiopathology of Obesity and Nutrition, ISCIII, Santiago de Compostela, Spain; 4 Laboratory of Development and Plasticity of the Neuroendocrine Brain, Inserm, University of Lille, Lille, France; 5 Pediatrics Department, GI Pediatric Nutrition, Galicia Research Unit for Development, Growth and Human Nutrition, Complejo Hospitalario Universitario de Santiago, Santiago de Compostela, Spain; 6 CNIC, Madrid, Spain; 7 Institute for Experimental and Clinical Pharmacology and Toxicology, University of Lübeck, Lübeck, Germany; 8 IGF, University of Montpellier, CNRS, Inserm, Montpellier, France; 9 Galician Agency of Innovation (GAIN), Xunta de Galicia, Santiago de Compostela, Spain

**Keywords:** Obesity, Hypothalamus, Hypothalamus

## Abstract

Early-life determinants are thought to be a major factor in the rapid increase of obesity. However, while maternal nutrition has been extensively studied, the effects of breastfeeding by the infant on the reprogramming of energy balance in childhood and throughout adulthood remain largely unknown. Here we show that delayed weaning in rat pups protects them against diet-induced obesity in adulthood, through enhanced brown adipose tissue thermogenesis and energy expenditure. In-depth metabolic phenotyping in this rat model as well as in transgenic mice reveals that the effects of prolonged suckling are mediated by increased hepatic fibroblast growth factor 21 (FGF21) production and tanycyte-controlled access to the hypothalamus in adulthood. Specifically, FGF21 activates GABA-containing neurons expressing dopamine receptor 2 in the lateral hypothalamic area and zona incerta. Prolonged breastfeeding thus constitutes a protective mechanism against obesity by affecting long-lasting physiological changes in liver-to-hypothalamus communication and hypothalamic metabolic regulation.

## Abstract (web summary)

Extended exposure to breastfeeding in rodent pups protects them from diet-induced obesity in adulthood by increasing thermogenesis and energy expenditure. This is mediated by liver-derived FGF21 signalling to the lateral hypothalamic area.

## Main

Obesity, whose prevalence has increased over the last few decades to reach pandemic status, is a multifactorial pathology, influenced by environmental, genetic and epigenetic factors^1^. In keeping with the essential role that neonatal events are thought to play in neurodevelopment and behavioural responses later in adulthood, this rapid increase in the prevalence of obesity may also result from early-life determinants^2^. Among these determinants are maternal diet and neonatal feeding.

The effect of nutritional alterations in the dam during the lactation–suckling period on obesity programming in the offspring has been widely studied in animal models. Maternal high-fat diet (HFD) during lactation modifies the composition of maternal milk and predisposes the offspring to obesity and impaired glucose homeostasis in adulthood^3,4^. This metabolic dysfunction has been associated with an impairment of white adipose tissue (WAT) function^4,5^, an inhibition of the thermogenic activity of brown adipose tissue (BAT)^3^ and damaged hypothalamic circuits^6^.

In contrast, while animal studies have suggested that neonatal overfeeding is a critical parameter influencing long-term metabolic outcomes^7–9^, studies concerning the influence of feeding/suckling by the offspring themselves on the long-term reprogramming of energy balance have been inconclusive so far. Some epidemiological studies have reported that breastfeeding has a protective effect against obesity in childhood/adulthood^10–12^. However, many of these studies are based on descriptive associations and limited by low sample size or confounding factors, while others have failed to find a correlation between breastfeeding and adiposity in children^13–17^. Nevertheless, breast milk is enriched in several bioactive factors that may be involved in the modulation of different mechanisms controlling energy homeostasis. For instance, leptin ingested as a component of breast milk is recognized to play a role in the postnatal programming of a healthy phenotype in adulthood^18^, and the duration of breastfeeding is also associated with epigenetic alterations in the leptin gene in children^19^.

To determine whether the reprogramming of energy homeostasis by breastfeeding is transient or lasts into adulthood and what molecular factors could underlie these putative metabolic changes, we designed a model of prolonged breastfeeding or suckling in rats, followed by feeding with chow diet (CD) or HFD until adulthood. With the aid of in-depth metabolic phenotyping in this model as well as in transgenic mice, and using local or systemic injections of metabolic hormones or the local knockdown of molecules involved in their signalling, we investigated the peripheral and central effects of prolonged suckling on HFD-induced long-term changes. Our data show not only how prolonged suckling modifies metabolic parameters in offspring subjected to a normal diet or HFD, but also elucidate the hypothalamic mechanisms triggered by peripheral signals to effect these changes in energy balance.

## Results

### Prolonged suckling reduces high-fat diet-induced weight gain

**Fig. 1:**
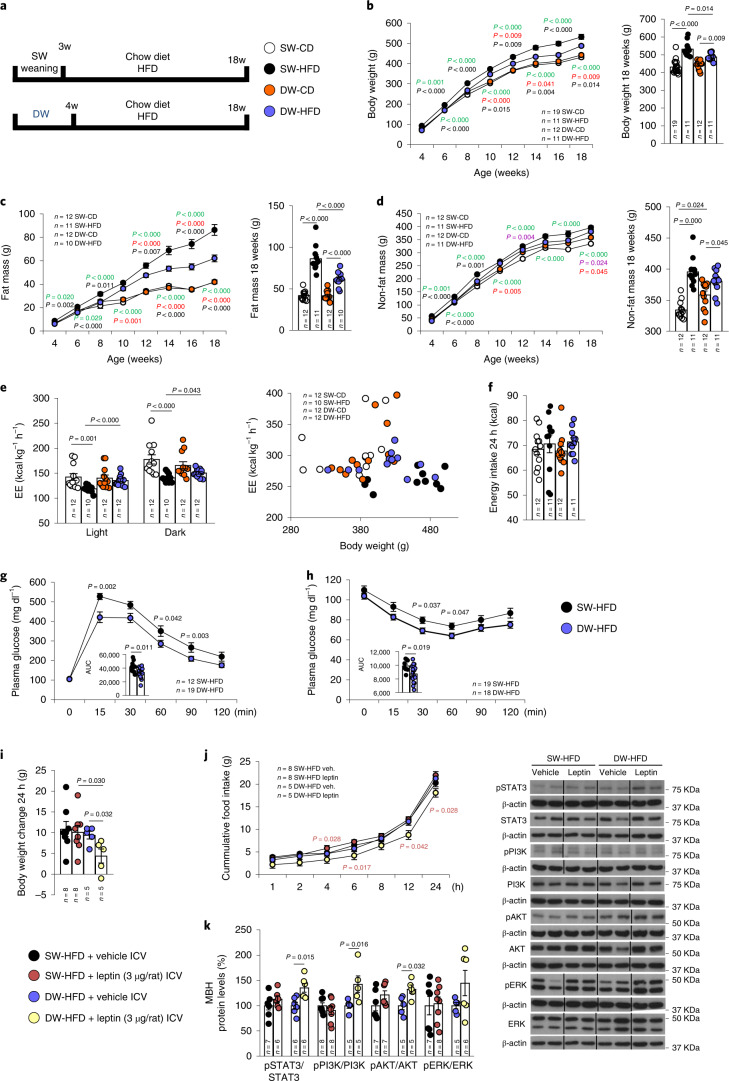
Prolonged suckling (delayed weaning) decreases body weight and fat mass while increasing energy expenditure, with no change of food intake in rats. **a**, Timeline of the experimental protocol. **b**–**h**, Effects of delayed weaning on body weight (**b**); fat mass (**c**); non-fat mass (**d**); energy expenditure (EE) in light and dark phases and as a function of body weight (**e**); energy intake over 24 h (**f**); glucose tolerance (**g**); and insulin response (**h**). **i**–**k**, In rats fed an HFD, effect of delayed weaning on response to ICV administration of leptin (3 μg per rat) in terms of body weight change (**i**), cumulative food intake (**j**) and MBH protein levels of pSTAT3, STAT3, pPI3K, PI3K, pAKT, AKT, pERK and ERK (**k**). Protein data were expressed as percentages in relation to control (SW-HFD vehicle) animals. β-actin was used to normalize protein levels. Dividing lines indicate splicing within the same gel. Values are represented as means ± s.e.m., *n* per group. Exact *P* values are shown. Statistical differences between groups are indicated by the following colours: black, SW-HFD versus DW-HFD; green, SW-CD versus SW-HFD; violet, SW-CD versus DW-CD; red, DW-CD versus DW-HFD. Statistical differences were determined by one-way analysis of variance (ANOVA; normal data and homogeneity of variances) followed by Tukey’s post hoc multiple-comparison test (**b**–**d**, **f** and **j**) or a two-sided Student’s *t*-test (normal data; **g** and **h**), a two-sided Mann–Whitney *U* test (non-normal data and non-homogeneous variance; **e**, **i** and **k**), or an analysis of covariance (ANCOVA) with body weight as a covariate (**e**). AUC, area under the curve. Source data

To determine whether extended suckling in rats, modelling prolonged breastfeeding in human infants, could exert a long-term influence on the management of energy intake, litters born to chow-fed dams were weaned either at 3 weeks after birth (postnatal day 21, standard weaning or SW) or at 4 weeks (delayed weaning or DW). SW and DW pups were subsequently maintained on a CD or HFD until they were 18 weeks old (Fig. 1a).

**Extended Data Fig. 1:**
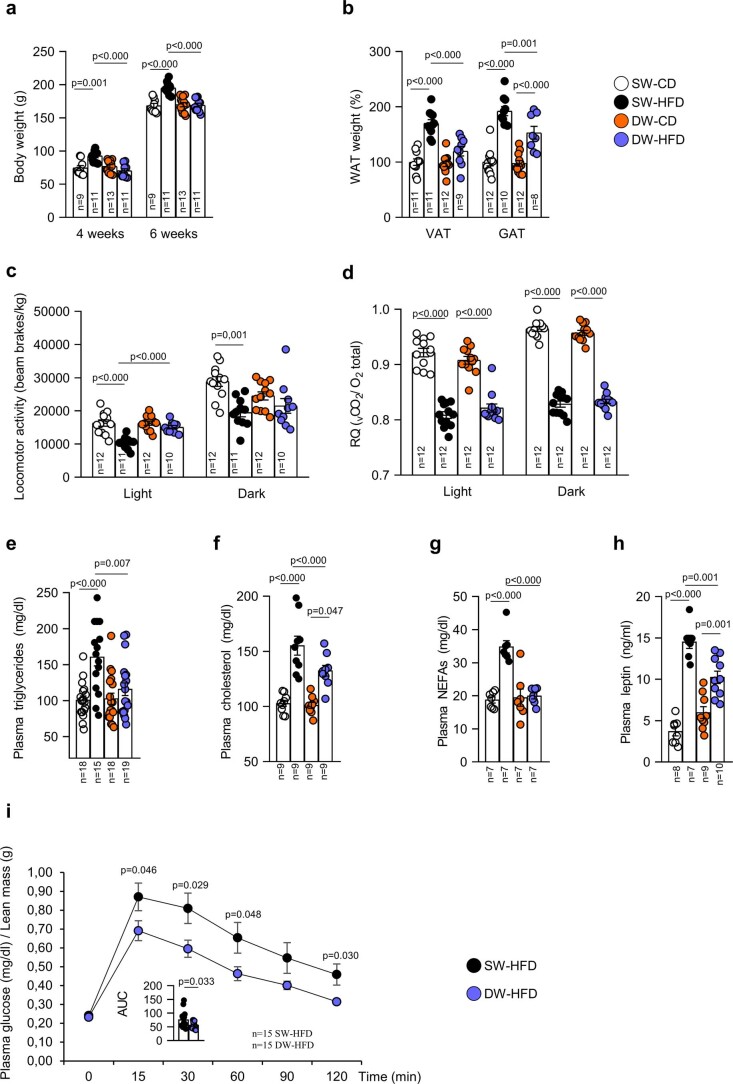
Prolonged breastfeeding decreases fat mass and improves dyslipidemia. **a**–**h**, Delayed weaning effects on body weight (**a**), white adipose tissue (WAT) weight in terms of the weight of Visceral Adipose Tissue (VAT) and Gonadal Adipose Tissue (GAT) (n = 8–12) (**b**); locomotor activity (**c**); respiratory quotient (RQ) (**d**); circulating levels of triglycerides (**e**), cholesterol (**f**), non-esterified fatty acids (NEFAs) (**g**), and leptin (**h**). **i**, Delayed weaning effects on glucose tolerance normalized with lean mass. Values are represented as means ± SEM, n per group indicated in each figure. Exact P values are shown. Statistical differences according to a One-way analysis of variance ANOVA (normal data and homogeneity of variances) followed by Tukey’s post hoc multiple comparison test (**a**, **b**, **c**, **d**, **g** and **h**) or to a two-sided Student’s t-test (normal data) (**i**), a two-sided Mann-Whitney U-test (non-normal data and non-homogeneous variance) (**e** and **f**). Source data

Rats subjected to DW and given a chow diet (DW-CD) did not differ in terms of body weight from those subjected to SW and the same diet (SW-CD; Fig. 1b and Extended Data Fig. 1a). However, when DW rats were fed an HFD (DW-HFD), their body weight was substantially lower than that of SW animals that were kept on an HFD (SW-HFD) for the same period of time (Fig. 1b and Extended Data Fig. 1a).

In keeping with these results, an analysis of body composition using quantitative nuclear magnetic resonance (NMR) revealed that SW-CD and DW-CD rats had similar fat and non-fat mass values (Fig. 1c,d), but DW-HFD rats accumulated notably less fat mass than SW-HFD animals (Fig. 1c), while non-fat mass was unchanged between the two HFD groups (Fig. 1d).

Similar results were obtained when visceral and gonadal adipose tissues (VAT and GAT) were weighed (Extended Data Fig. 1b). In accordance with the lower fat content, energy expenditure was also higher in DW-HFD than in SW-HFD rats in both light and dark phases (Fig. 1e). These changes in energy expenditure between SW-HFD and DW-HFD rats were not associated with differences in locomotor activity, respiratory quotient (Extended Data Fig. 1c,d) or food intake (Fig. 1f). DW-HFD rats also showed reduced circulating levels of triglycerides, cholesterol, non-esterified fatty acids and leptin (Extended Data Fig. 1e–h), as well as improved glucose tolerance and enhanced insulin sensitivity when compared to SW-HFD animals (Fig. 1g,h and Extended Data Fig. 1i).

**Fig. 2:**
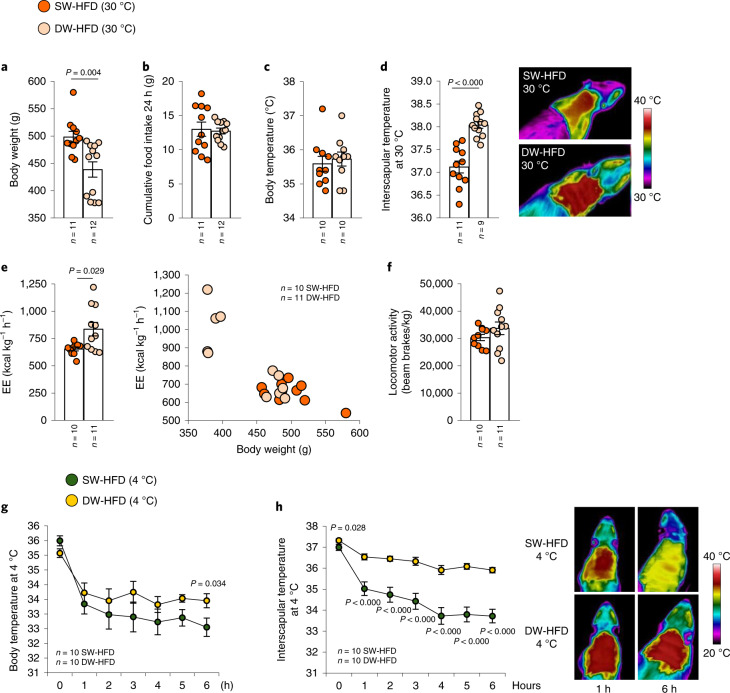
Prolonged suckling (delay weaning) increases interscapular temperature in both thermoneutral and cold exposureconditions. **a**–**f**, Delayed-weaning effects in rats fed an HFD under thermoneutral conditions on body weight (**a**); cumulative food intake (**b**); body temperature (**c**); infrared thermal images and quantification of BAT interscapular temperature (**d**); energy expenditure (**e**) and locomotor activity (**f**). **g**,**h**, Delayed-weaning effects in rats fed an HFD and exposed to cold (4 °C) for 6 h on body temperature (**g**) and infrared thermal images and quantification of BAT interscapular temperature at 4 °C (**h**). Values are represented as means ± s.e.m., *n* per group. Exact *P* values are shown. Statistical differences were determined by a two-sided Student’s *t*-test (normal data; **c**–**h**), a two-sided Mann–Whitney *U* test (non-normal data and non-homogeneous variance; **a** and **b**), or an ANCOVA with body weight as a covariate (**e**). Source data

Next, we evaluated the phenotype of SW-HFD and DW-HFD rats in a thermoneutral environment (30 °C) to eliminate the extra metabolism necessary to maintain body temperature at lower ambient temperatures. Consistent with the results above, body weight was reduced while food intake was unchanged in DW-HFD rats (Fig. 2a,b). However, while body temperature was similar between the two groups (Fig. 2c), DW-HFD rats displayed a higher interscapular temperature than SW-HFD rats (Fig. 2d). Under thermoneutral conditions, energy expenditure was higher in DW-HFD rats, without any difference in locomotor activity (Fig. 2e,f). We then conducted a cold-exposure test to assess the thermogenic response of BAT and found that DW-HFD rats preserved their whole-body and interscapular temperatures better than SW-HFD rats at 4 °C (Fig. 2g,h).

**Extended Data Fig. 2:**
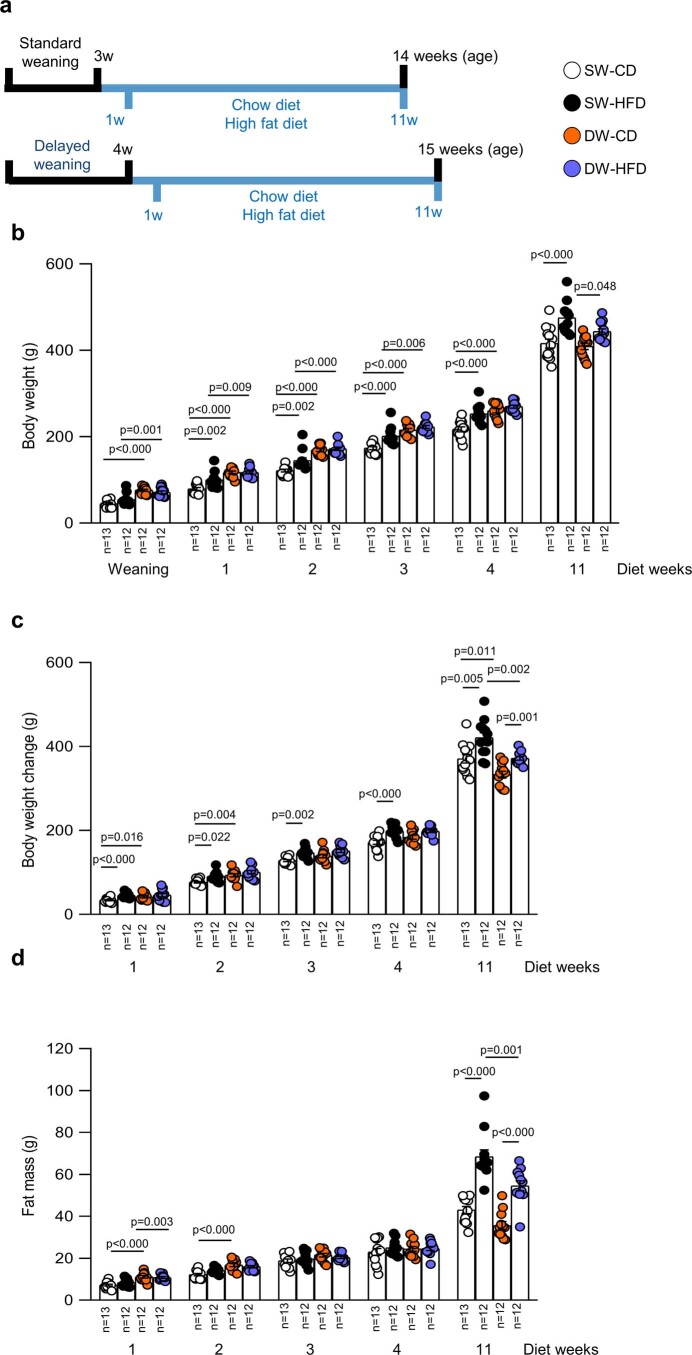
Prolonged breastfeeding protects against diet-induced obesity at early stages. Prolonged suckling (delayed weaning) decreases body weight and fat mass in rats accordingly to the weeks of exposition to high fat diet (HFD) instead of weeks of age. **a**, Timeline of the experimental protocol. **b**–**d**, Effects of delayed weaning on body weight (**b**), body weight changes (**c**) and fat mass (**d**). Values are represented as means ± SEM, n per group indicated in each figure. Exact P values are shown. Statistical differences according to a One-way analysis of variance ANOVA (normal data and homogeneity of variances) followed by Tukey’s post hoc multiple comparison test (**b**, **c** and **d**). Source data

It should be noted that, an extra week of HFD feeding is present in the SW-HFD group. To investigate whether the extra week of HFD could have implications in the observed phenotype, the body weight data were also reanalysed by comparing the body weights of SW-HF and DW-HFD groups accordingly to the weeks of exposure to an HFD instead of weeks of age. It was found that DW-HFD rats still presented lower body weight gain, and lower fat mass than SW-HFD rats (Extended Data Fig. 2). Altogether, these findings indicate that prolonged suckling increases the thermogenic activity of BAT in HFD offspring, increasing energy expenditure and ultimately reducing weight gain in a feeding-independent fashion.

### Prolonged suckling increases leptin sensitivity in high-fat diet-fed rats

To evaluate whether prolonged breastfeeding could improve leptin sensitivity in obesity, both SW-HFD and DW-HFD rats were given an intracerebroventricular (ICV) injection of either vehicle or leptin at a dose of 3 µg, known to induce a decrease in feeding and body weight in CD-fed rodents^20^.

Leptin ICV injection did not induce any change in body weight (Fig. 1i) or food intake (Fig. 1j) in SW-HFD animals, in keeping with the leptin resistance observed in animals and humans with diet-induced obesity (DIO)^21,22^. In contrast, when administered to DW-HFD animals, leptin significantly decreased body weight (Fig. 1i) and food intake over 24 h (Fig. 1j). After collecting the hypothalamus of the different groups of rats, we assessed the impact of DW on the hypothalamic leptin receptor signalling pathway, expected to be impaired in HFD rats. In the mediobasal hypothalamus (MBH) of DW-HFD rats, but not SW-HFD animals, leptin increased the ratios of pSTAT3/STAT3, pPI3K/PI3K, pAKT/AKT and pERK/ERK (Fig. 1k), indicating the activation of leptin receptor signalling. Altogether, these results indicate that prolonged suckling enhances the responsiveness to leptin of HFD-fed rats.

### Prolonged suckling activates brown adipose tissue thermogenesis

**Fig. 3:**
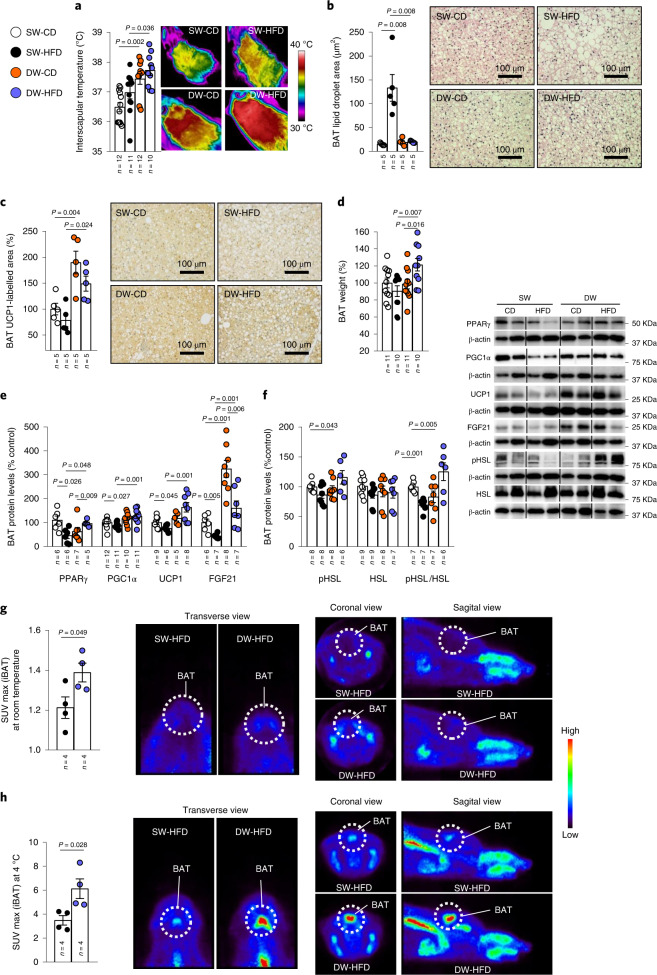
Prolonged suckling activates BAT thermogenesis and browning of WAT. **a**–**h**, Effects of delayed weaning shown on infrared thermal images and quantification of BAT interscapular (iBAT) temperature (*n* = 10–12; **a**); quantification of lipid droplet cross-sectional area in BAT (*n* = 5; **b**); quantification of immunolabelling for UCP1 in BAT (*n* = 5; **c**); BAT weight (*n* = 10–11; **d**); BAT protein levels of PPARγ, PGC1α, UCP1 and FGF21 (*n* = 5–12; **e**) and pHSL, HSL and pHSL/HSL (*n* = 6–9; **f**); ^18^F-FDG uptake analysis (standardized uptake value (SUV max)) of rats at room temperature (*n* = 4; **g**); and ^18^F-FDG uptake analysis of rats at 4 °C (*n* = 4; **h**). Protein data were expressed as percentages in relation to control (SW-CD) animals. β-actin was used to normalize protein levels. Dividing lines indicate splicing within the same gel. Values are represented as means ± s.e.m., *n* per group indicated in each figure. Exact *P* values are shown. Statistical differences were determined by one-way ANOVA (normal data and homogeneity of variances) followed by Tukey’s post hoc multiple-comparison test (**a** and **c**) or a two-sided Student’s *t*-test (normal data; **g** and **h**) or a two-sided Mann–Whitney *U* test (non-normal data and non-homogeneous variance; **b** and **d**–**f**). Source data

In keeping with the lower body weight and increased energy expenditure seen in DW-HFD rats, these animals also displayed increased interscapular temperature and weight of BAT with respect to the SW-HFD group (Fig. 3a). Furthermore, histological analysis revealed smaller lipid droplets in the BAT of this group, comparable to those in CD-fed animals (Fig. 3b). In agreement with this, BAT weight and immunolabelling for uncoupling protein 1 (UCP1), the main BAT thermogenesis marker, were higher in DW rats fed with either a CD or HFD than in SW rats (Fig. 3c,d). Consistent with the histological data, protein levels of UCP1 were elevated in DW-HFD compared to SW-HFD rats, and the same pattern was observed for other proteins such as peroxisome proliferator-activated receptor-γ coactivator-1-α (PGC1α), peroxisome proliferator-activated receptor-γ (PPARγ) and FGF21, which are predictive of thermogenesis^23^ (Fig. 3e). In addition, protein levels of phosphorylated hormone-sensitive lipase (pHSL), a lipolytic marker, and the ratio of pHSL/HSL was also increased in the BAT of DW-HFD rats (Fig. 3f).

In agreement with the increased BAT thermogenesis, DW-HFD animals showed increased glucose uptake in the BAT at room temperature (Fig. 3g) and after cold exposure (Fig. 3h), as indicated by ^18^F-fluoro-2-deoxy-d-glucose (^18^F-FDG) uptake in BAT. The data from positron emission tomography-computed tomography (PET/CT) confirmed the activation of the thermogenic programme elicited by prolonged breastfeeding in animals fed an HFD.

**Extended Data Fig. 3:**
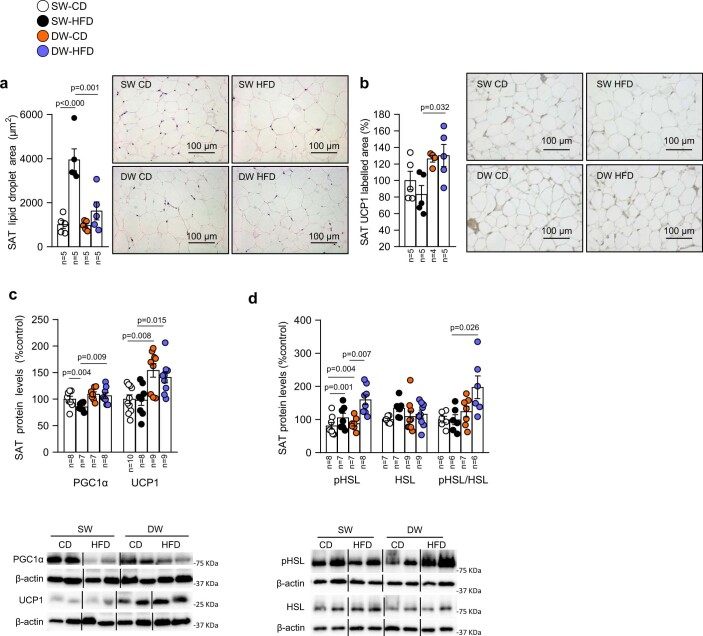
Prolonged breastfeeding reduces lipid droplets and increases UCP1 in adipose tissue. Prolonged suckling activates browning of WAT. **a**–**d**, Effects of delayed weaning on quantification of lipid droplet cross-sectional area in SAT (**a**); quantification of immunolabeling for UCP1 in SAT (**b**); SAT protein levels of PGC1α and UCP1 (**c**); SAT protein levels of pHSL, HSL and ratio pHSL/HSL (**d**). Protein data were expressed in relation (%) to control (SW-CD) animals. β-actin was used to normalize protein levels. Dividing lines indicate splicing within the same gel. Values are represented as means ± SEM, n per group indicated in each figure. Exact P values are shown. Statistical differences according to a One-way analysis of variance ANOVA (normal data and homogeneity of variances) followed by Tukey’s post hoc multiple comparison test (**a** and **b**) or to a two-sided Mann-Whitney U-test (non-normal data and non-homogeneous variance) (**c** and **d**). Source data

Given the activation of the thermogenic programme in the BAT of DW-HFD rats, we then assessed the potential thermogenic role of browning in WAT. The lipid content of the subcutaneous adipose tissue (SAT), as measured by the area covered by lipid droplets, was decreased in DW-HFD compared to SW-HFD rats (Extended Data Fig. 3a). Accordingly, immunolabelling for UCP1 and protein levels of both UCP1 and PGC1α were increased in DW-HFD rats (Extended Data Fig. 3b,c), indicating an increase in browning of WAT. In addition, the pHSL/HSL ratio, a surrogate marker of lipolysis, was also upregulated in DW-HFD rats (Extended Data Fig. 3d).

**Extended Data Fig. 4:**
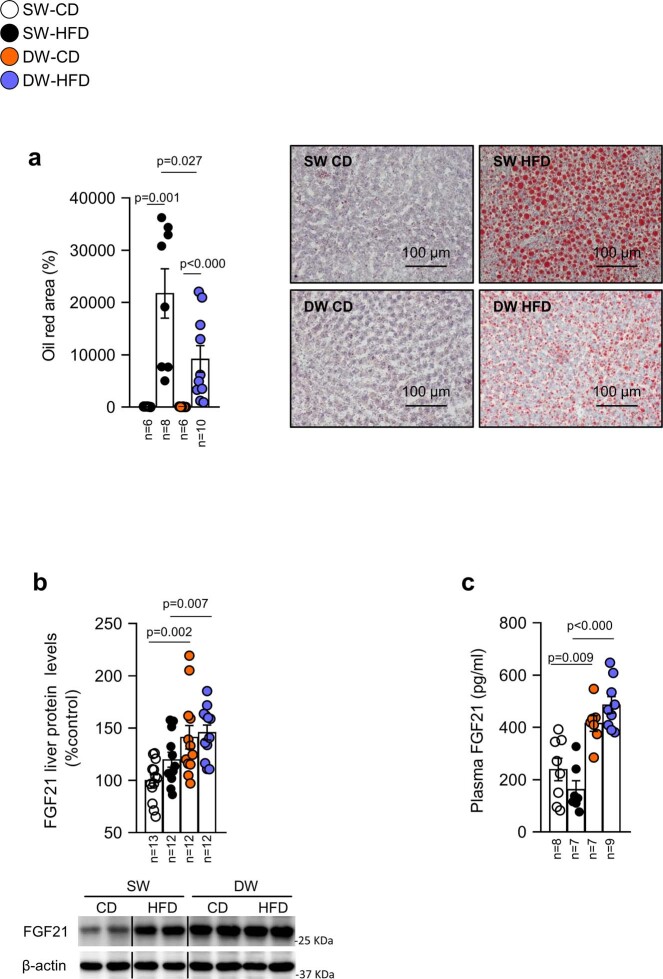
Prolonged breastfeeding reduces liver steatosis and stimulates FGF21 levels. Prolonged suckling alleviates HFD-induced steatosis. **a**–**c**, Effects of delayed weaning on oil red area in the liver (**a**); liver protein levels of FGF21 (**b**); plasma FGF21 levels (**c**). Protein data were expressed in relation (%) to control (SW-CD) animals. β-actin was used to normalize protein levels. Dividing lines indicate splicing within the same gel. Values are represented as means ± SEM, n per group indicated in each figure. Exact P values are shown. Statistical differences according to a One-way analysis of variance ANOVA (normal data and homogeneity of variances) followed by Tukey’s post hoc multiple comparison test (**c**) or to a two-sided Mann-Whitney U-test (non-normal data and non-homogeneous variance) (**a** and **b**). Source data

Interestingly, hepatic steatosis, a hallmark commonly observed in DIO animals, was also ameliorated in DW-HFD rats (Extended Data Fig. 4a). We therefore measured levels of FGF21, a hepatokine with a potent capacity to stimulate thermogenic activity^23^ and reduce hepatic steatosis^24^. Hepatic and plasma levels of FGF21 were significantly increased in DW rats (Extended Data Fig. 4b,c), suggesting that FGF21, which is predominantly produced by the liver, might play a role in reversing or preventing HFD-induced changes in animals suckled for prolonged periods.

**Extended Data Fig. 5:**
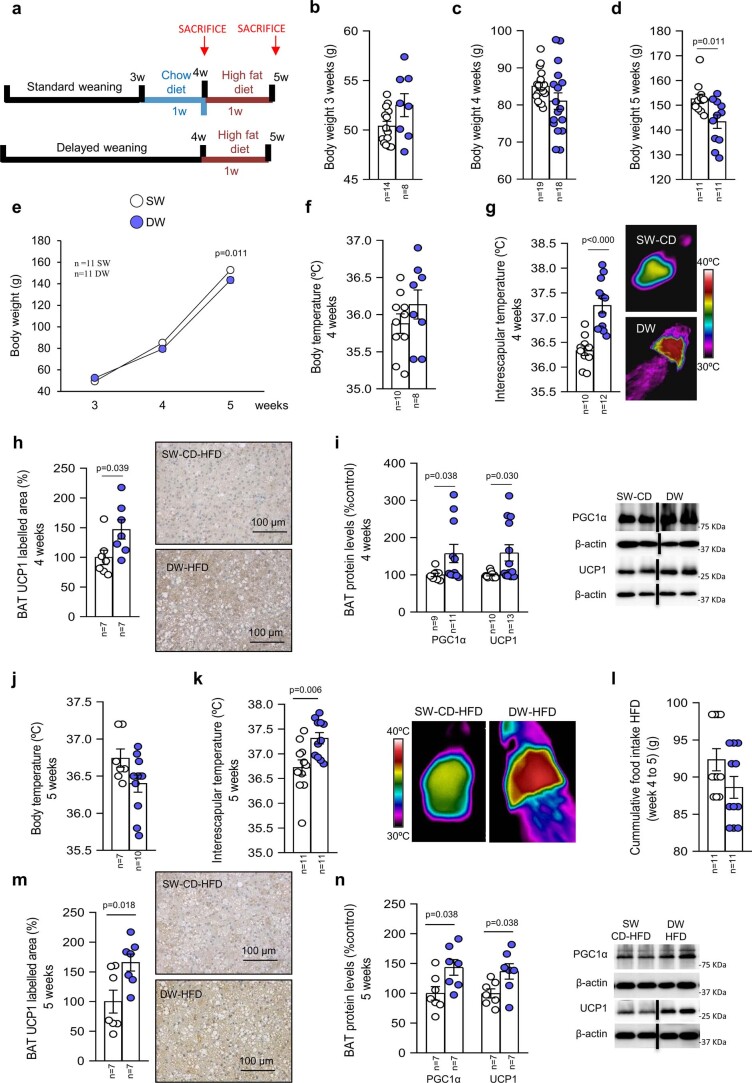
Prolonged breastfeeding activates brown fat thermogenesis after weaning. Prolonged suckling (delayed weaning) decreases body weight while activates BAT thermogenesis in rats with only one week of HFD. **a**, Timeline of the experimental protocol. **b**–**n**, Effects of delayed weaning on body weight at 3 weeks (**b**); body weight at 4 weeks (**c**); body weight at 5 weeks (**d**); body weight change (**e**), body temperature at 4 weeks (**f**), infrared thermal images and quantification of BAT interscapular temperature at 4 weeks (**g**); quantification of immunolabeling for UCP1 in BAT at 4 weeks (**h**); BAT protein levels of PGC1α and UCP1 at 4 weeks (**i**); body temperature at 5 weeks (**j**), infrared thermal images and quantification of BAT interscapular temperature at 5 weeks (**k**); cumulative food intake HFD between week 4 and 5 (**l**); quantification of immunolabeling for UCP1 in BAT at 5 weeks (**m**); BAT protein levels of PGC1α and UCP1 at 5 weeks (**n**). Protein data were expressed in relation (%) to control (SW) animals. β-actin was used to normalize protein levels. Dividing lines indicate splicing within the same gel. Values are represented as means ± SEM, n per group indicated in each figure. Exact P values are shown. Statistical differences according to a two-sided Student’s t-test (normal data) (**b**, **d**, **e**, **f**, **h**, **j**, **k**, **l** and **m**), a two-sided Mann-Whitney U-test (non-normal data and non-homogeneous variance) (**c**, **g**, **i** and **n**). Source data

To avoid the possible effect of the extra week of HFD feeding in the SW-HFD model on thermogenesis, an additional short experiment was performed. After the weaning, SW-HFD rats were fed a CD for 1 week, and then were exposed to an HFD for another week (Extended Data Fig. 5a). So, in this model, both groups (SW and DW) were exposed to 1 week of HFD feeding, and the only difference among both groups was the additional exposure to breastfeeding for 1 week in the DW group (Extended Data Fig. 5a). DW-HFD rats presented lower body weight, higher interscapular temperature and increased expression of the thermogenic markers UCP1 and PGC1a in BAT (Extended Data Fig. 5b–n). It is also important to note that at 4 weeks of age, after the introduction of HFD, although the body weight of both DW and SW was similar, the interscapular temperature was already increased, indicating that the effects of breastfeeding on BAT thermogenesis precede differences in body weight.

### Hepatic FGF21 knockdown prevents prolonged suckling effects

**Fig. 4:**
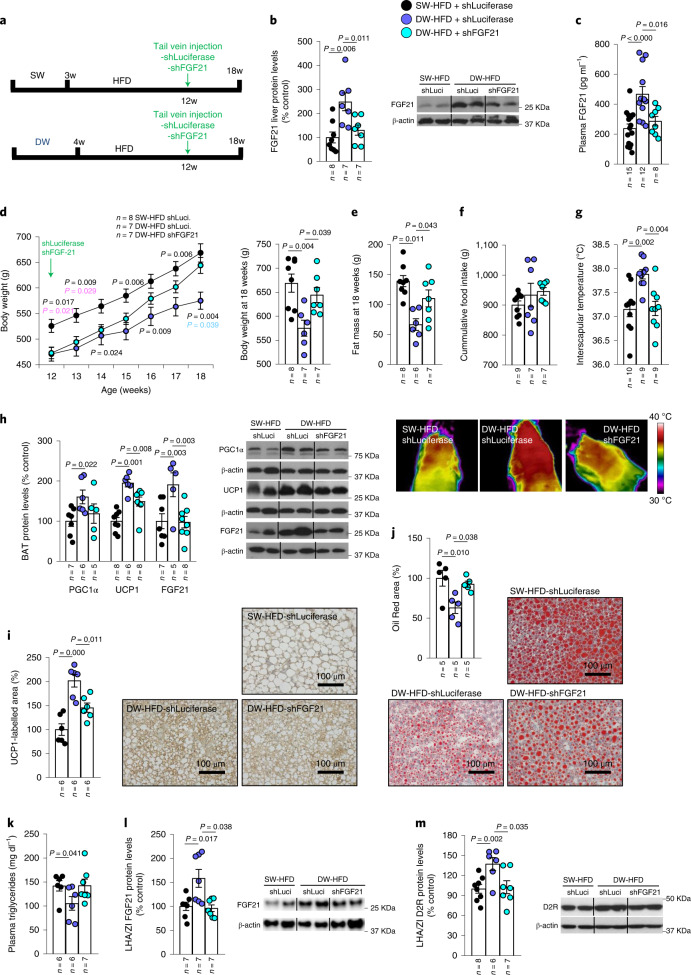
Knockdown of *Fgf21* in the liver blunts delayed-weaning-induced weight loss. **a**, Timeline of the experimental protocol. **b**–**m**, Effect of infection with adenoviral particles encoding sh*Fgf21* in the tail vein of 12-week-old rats fed an HFD after prolonged suckling on liver protein levels of FGF21 (**b**); plasma FGF21 levels (**c**); body weight (**d**); fat mass (**e**); cumulative food intake (**f**); infrared thermal images and quantification of BAT interscapular temperature (**g**); BAT protein levels of PGC1α, UCP1 and FGF21 (**h**); quantification of immunolabelling for UCP1 in BAT (**i**); Oil Red area in the liver (**j**); plasma triglycerides (**k**); LHA/ZI protein levels of FGF21 (**l**); and LHA/ZI protein levels of D2R (**m**). Protein data were expressed as percentages in relation to control (SW-HFD shLuciferase) animals. β-actin was used to normalize protein levels. Dividing lines indicate splicing within the same gel. Values are represented as means ± s.e.m., *n* per group. Exact *P* values are shown. Statistical differences were determined by one-way ANOVA (normal data and homogeneity of variances) followed by Tukey’s post hoc multiple-comparison test (**c**–**e**, **g** and **i**–**k**) or a two-sided Mann–Whitney *U* test (non-normal data and non-homogeneous variance; **b**, **f**, **h**, **l** and **m**). Source data

To determine whether the increased hepatic FGF21 production demonstrated above was causally linked to the effects of prolonged suckling on DIO, *Fgf21* was reduced by injecting a lentiviral vector encoding an shRNA against *Fgf21* into the tail vein of DW-HFD rats (Fig. 4a), a technique previously shown to be effective in selectively targeting the liver^25^. Lentiviral shRNA delivery normalized the increased hepatic expression and circulating levels of FGF21 observed in DW-HFD rats to SW-HFD levels (Fig. 4b,c). While DW-HFD rats gained less weight than their controls, as seen above (Fig. 1b), the hepatic knockdown of FGF21 partially blunted this effect (Fig. 4d) as well as the decrease in adiposity (Fig. 4e), without affecting feeding behaviour (Fig. 4f). In addition, the increased interscapular temperature displayed by DW-HFD rats was also reversed when hepatic FGF21 was reduced (Fig. 4g). In accordance with the decreased interscapular temperature, the reduction in FGF21 levels also reversed the increased expression of the BAT thermogenesis markers PGC1α and UCP1 induced by prolonged suckling (Fig. 4h) as well as UCP1 immunolabelling in BAT (Fig. 4i). In addition, the reduction of hepatic *Fgf21* reversed the increased FGF21 levels in BAT (Fig. 4h). The knockdown of *Fgf21* in DW-HFD rats also reversed the reduction of lipid accumulation in the liver, as shown by Oil Red O staining (Fig. 4j) and lowered circulating triglycerides (Fig. 4k). Together, these results indicate that the beneficial effects of prolonged suckling are mediated by an increase in liver FGF21 production.

**Extended Data Fig. 6:**
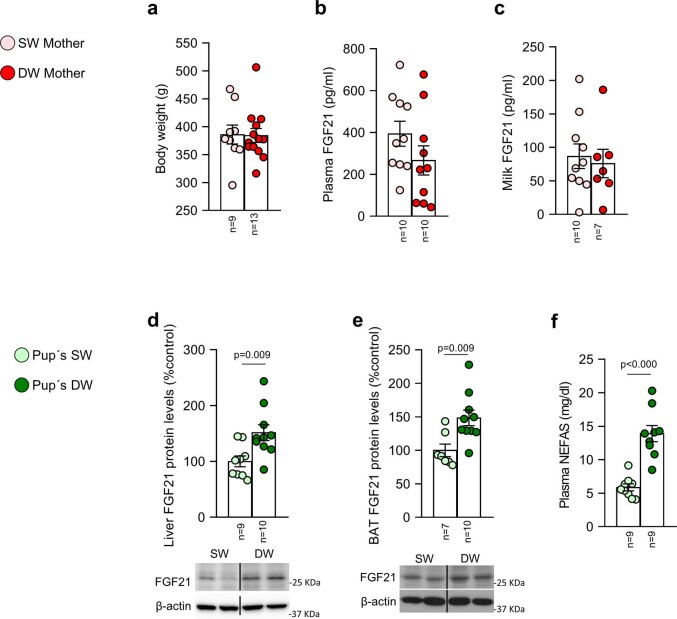
FGF21 is increased in pups but not mothers after prolonged breastfeeding. The different levels observed in FGF21 between SW-HFD and DW-HFD animals are not coming from the mothers. **a**–**c**, Effects of delayed weaning on mother´s: body weight (**a**), plasma FGF21 levels (**b**), milk FGF21 levels. **d**–**f**, Effects of delayed weaning on pups: liver protein levels of FGF21 (n = 9–10) (**d**); BAT protein levels of FGF21 (**e**); plasma NEFAS (**f**). Protein data were expressed in relation (%) to control (SW) animals. β-actin was used to normalize protein levels. Dividing lines indicate splicing within the same gel. Values are represented as means ± SEM, n per group indicated in each figure. Exact P values are shown. Statistical differences according to a two-sided Student’s t-test (normal data) (**a**, **b**, **c**, **d**, **e** and **f**). Source data

The next step was to elucidate why prolonged suckling increased plasma concentrations of FGF21. To test if the increased FGF21 levels in the offspring were a consequence of the transfer of maternal FGF21 through the milk, FGF21 levels were analysed in plasma and milk from the two different groups of mothers—those nursing the animals for the standard period and mothers of animals subjected to delayed weaning. The body weight of the two groups of mothers was the same at weaning (Extended Data Fig. 6a), and FGF21 levels in plasma or milk were also similar (Extended Data Fig. 6b,c). These findings indicated that the different levels observed in FGF21 were not due to an increased transference of FGF21 from prolonged suckling mothers to the pups.

We measured protein levels of FGF21 in the liver (Extended Data Fig. 6d) and BAT (Extended Data Fig. 6e) of pups and found that they were augmented just after weaning in those animals after extended suckling compared to those suckling for a normal period. Thus, the results indicated that the additional week of breastfeeding was increasing the production of FGF21 levels in the pups. Because fatty acids were proposed as the main regulators of FGF21 expression^23^, we measured circulating non-esterified fatty acids and found that their levels were higher in those animals under prolonged breastfeeding (Extended Data Fig. 6f).

### Brain dopaminergic D2 receptor mediates the effects of prolonged suckling

Energy metabolism and feeding behaviour are regulated by hypothalamic neurons, which respond to circulating metabolic signals^26–29^.

Interestingly, we have recently described a hypothalamic mechanism regulating BAT thermogenesis that involves the expression of the dopaminergic D2 receptor (D2R) specifically in the lateral hypothalamic area (LHA) and zona incerta (ZI) but no other hypothalamic regions^30^. To test whether this dopaminergic mechanism could be involved in the FGF21-dependent metabolic effects of prolonged suckling, we measured the protein levels of FGF21 and D2R in the LHA/ZI.

**Extended Data Fig. 7:**
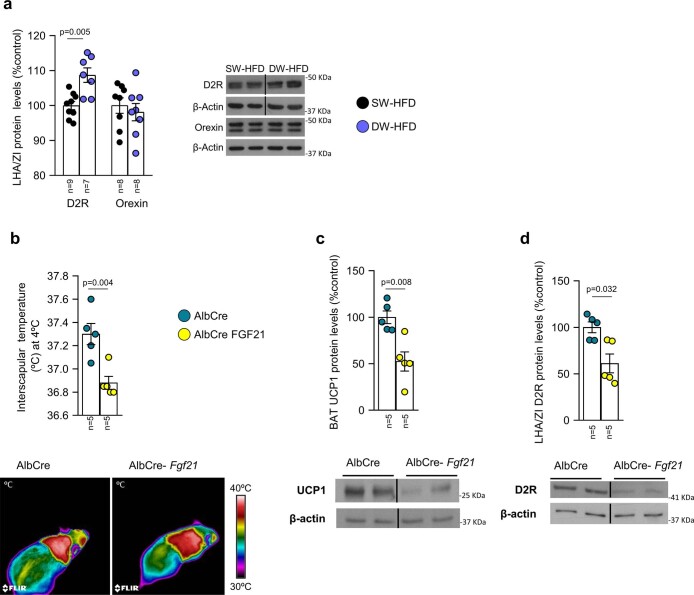
FGF21 increases protein levels of D2R in the LHA/ZI. **a**, Delayed weaning effects on LHA/ZI protein levels of D2R and Orexin levels in rats fed HFD. **b**–**d**, Effect of exposure to cold in mice lacking FGF21 in the liver (Albcre) on infrared thermal images and quantification of BAT interscapular temperature (**b**); BAT protein levels of UCP1 (**c**); LHA/ZI protein levels of D2R (**d**). Protein data were expressed in relation (%) to control (SW) animals. β-actin was used to normalize protein levels. Dividing lines indicate splicing within the same gel. Values are represented as means ± SEM, n per group indicated in each figure. Exact P values are shown. Statistical differences according to a two-sided Student’s t-test (normal data) (**b**), a two-sided Mann-Whitney U-test (non-normal data and non-homogeneous variance) (**a**, **c** and **d**). Source data

Indeed, DW-HFD rats showed increased levels of FGF21 in the LHA/ZI compared with SW-HFD animals, which were blunted when hepatic *Fgf21* was knocked down (Fig. 4l). Intriguingly, D2R levels, but not orexin, were also increased in the LHA/ZI of DW-HFD compared with SW-HFD animals (Extended Data Fig. 7a) but downregulated again when hepatic *Fgf21* production was blocked (Fig. 4m). Overall, these results support the view that FGF21 overproduction during extended suckling reaches hypothalamic dopaminergic circuits to mediate increased BAT thermogenesis.

Reconfirming the relevance of hepatic *Fgf21* on D2R expression in the LHA/ZI, mice lacking *Fgf21* in the liver, which showed lower interscapular temperature and lower levels of UCP1 in BAT when exposed to cold (Extended Data Fig. 7b,c), also displayed lower protein levels of D2R in the LHA/ZI (Extended Data Fig. 7d).

### FGF21 administration mimics prolonged suckling effects

**Fig. 5:**
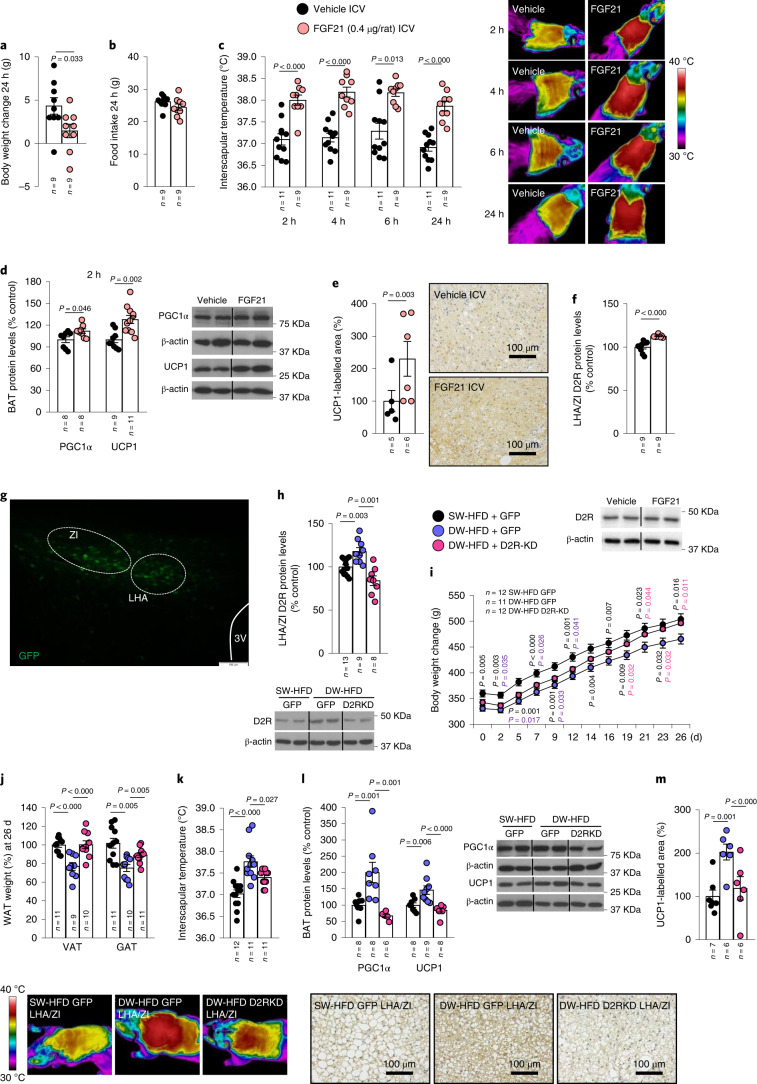
FGF21 decreases body weight by increasing thermogenesis in BAT and D2R expression in LHA/ZI, while knockdown of D2R in the LHA/ZI blunts delayed-weaning-induced weight loss. **a**–**f**, Effect of ICV injection of FGF21 (0.4 µg per rat) in male rats fed a CD on body weight change at 24 h (**a**); food intake at 24 h (**b**); infrared thermal images and quantification of BAT interscapular temperature at 2, 4, 6 and 24 h (**c**); BAT protein levels of PGC1α and UCP1 (**d**); quantification of immunolabelling for UCP1 in BAT (**e**) and LHA/ZI protein levels of D2R (**f**). **g**, Representative photomicrograph of a brain section showing GFP expression following injection of viral vectors that encode GFP expression precisely into the LHA/ZI; scale bar, 0.1 mm. **h**–**m**, Effect of injecting adenoviral particles encoding GFP or shD2R (leading to D2R knockdown; KD) into the LHA/ZI of rats fed an HFD following prolonged suckling on LHA/ZI protein levels of D2R (**h**); body weight change (**i**); WAT weight in terms of weight of VAT and GAT (**j**); infrared thermal images and quantification of BAT interscapular temperature (**k**); BAT protein levels of PGC1α and UCP1 (**l**); and quantification of immunolabelling for UCP1 in BAT (**m**). Protein data were expressed as percentages in relation to control (vehicle) animals. β-actin was used to normalize protein levels. Dividing lines indicate splicing within the same gel. Values are represented as means ± s.e.m., *n* per group. Exact *P* values are shown. Statistical differences were determined by one-way ANOVA (normal data and homogeneity of variances) followed by Tukey’s post hoc multiple-comparison test (**i** and **k**) or to a two-sided Student’s *t*-test (normal data; **a**–**c**), a two-sided Mann–Whitney *U* test (non-normal data and non-homogeneous variance; **d**–**f**, **h**, **j**, **l** and **m**). Source data

To further elucidate the effects of FGF21, we then tested the effects of centrally administered FGF21 on metabolic parameters and D2R expression in the LHA/ZI in lean control rats subjected to normal weaning and diet. ICV administration of FGF21 at a dose of 0.4 µg per rat reduced body weight after 24 h (Fig. 5a), an effect that was independent of food intake (Fig. 5b). FGF21 administration also induced a sustained increase in interscapular temperature after 2 h of its administration until at least 24 h, indicating increased BAT thermogenesis (Fig. 5c). Accordingly, PGC1α and UCP1 levels as well as UCP1 immunolabelling were increased in the BAT of rats that were administered ICV FGF21 (Fig. 5d,e). In addition, ICV FGF21 led to increased D2R expression in the LHA/ZI as expected (Fig. 5f).

### D2 receptor in LHA/ZI mediates prolonged suckling effects

**Extended Data Fig. 8:**
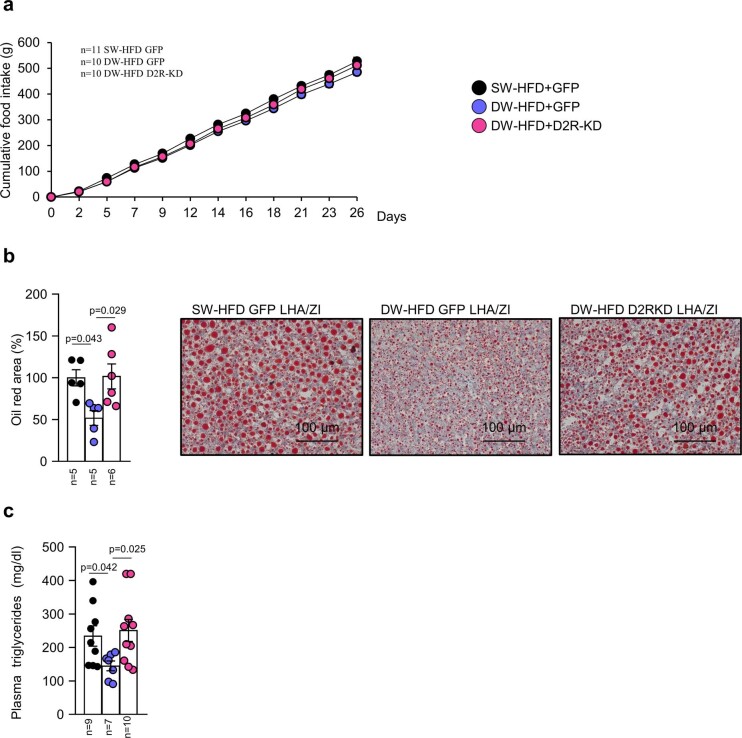
Knockdown of D2R in the LHA/ZI reverses the effects of prolonged breastfeeding in liver. **a**–**c**, Effect of injecting adenoviral particles encoding for GFP- or D2R-KD in the LHA/ZI of rats fed HFD with prolonged breastfeeding period on cumulative food intake (**a**); oil red area in Liver (**b**); plasma triglycerides (**c**). Values are represented as means ± SEM, n per group indicated in each figure. Exact P values are shown. Statistical differences according to a One-way analysis of variance ANOVA (normal data and homogeneity of variances) followed by Tukey’s post hoc multiple comparison test (**a**, **b**) or to a two-sided Mann-Whitney U-test (non-normal data and non-homogeneous variance) (**c**). Source data

Given our observation that D2R in the LHA/ZI is increased in DW-HFD animals (Fig. 4m) and induced by FGF21 administration (Fig. 5f), we next sought to determine whether this increase in LHA/ZI D2R levels contributed to the effect of extended suckling on HFD-induced weight gain. For this, *D2r* expression was reduced by injecting an adeno-associated virus (AAV) encoding an shRNA against *D2r* (sh*D2r*) or a scrambled sequence in addition to GFP into the LHA/ZI of DW-HFD rats^30^ (Fig. 5g). sh*D2r* treatment reduced D2R protein expression in the LHA/ZI of DW-HFD rats to levels similar to those in SW-HFD animals (Fig. 5h). It also prevented the reduction in weight gain and adiposity induced by DW in HFD rats (Fig. 5i,j), without affecting feeding behaviour (Extended Data Fig. 8a). Consistent with these changes, the stimulatory effects of prolonged suckling on interscapular temperature, protein levels of UCP1 and PGC1α and UCP1 immunolabelling in BAT were all partially or wholly blunted by *D2r* silencing in the LHA/ZI (Fig. 5k–m). In addition, the amelioration in liver steatosis and lower plasma triglycerides seen in DW-HFD rats was also reversed when *D2r* was reduced (Extended Data Fig. 8b,c). These results indicate that D2R in the LHA/ZI mediates the protective role of prolonged suckling against DIO.

### Central effects of FGF21 are dependent on D2 receptor in the LHA/ZI

**Fig. 6:**
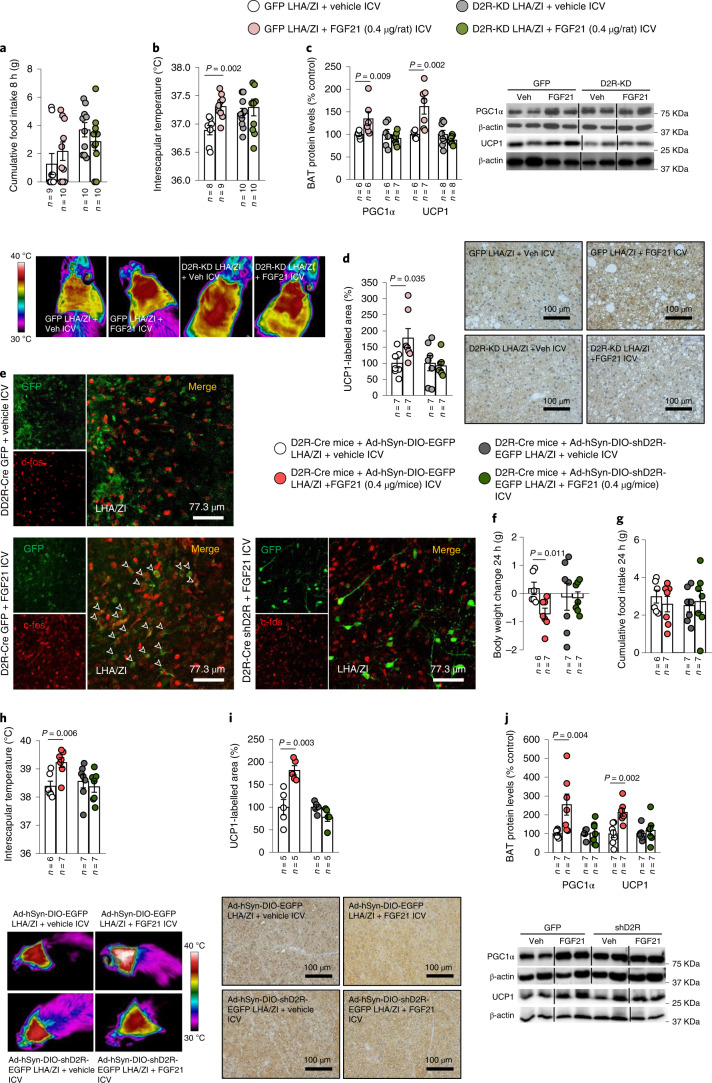
The effects of FGF21 on body weight and BAT thermogenesis are dependent on D2R in the LHA/ZI. **a**–**d**, Effect of injecting adenoviral particles encoding GFP or sh*D2r* (D2R-KD) into the LHA/ZI of rats fed a CD and treated with ICV FGF21 on cumulative food intake (**a**); infrared thermal images and quantification of BAT interscapular temperature (**b**); BAT protein levels of PGC1α and UCP1 (**c**); and quantification of immunolabelling for UCP1 in BAT (**d**). **e**, Photomicrographs showing the colocalization of GFP and c-Fos in the LHA/ZI of *D2r*-cre GFP mice treated with ICV FGF21, or shD2R + FGF21 ICV, demonstrating lack of c-Fos activation in LHA/ZI *D2r* neurons following *D2r* knockdown. **f**–**j**, Effect of injecting an adenoviral vector encoding a scrambled RNA (Ad-hSyn-DIO-EGFP) or an shRNA against *D2r* (Ad-hSyn-DIO-sh*D2r*-EGFP) in a Cre-dependent manner followed by ICV injection of vehicle or FGF21 into *D2r*-Cre mice on body weight change (**f**); cumulative food intake (**g**); infrared thermal images and quantification of BAT interscapular temperature (**h**); quantification of immunolabelling for UCP1 in BAT (**i**); and BAT protein levels of PGC1α and UCP1 (**j**). Protein data were expressed as percentages in relation to control (GFP vehicle) animals. β-actin was used to normalize protein levels. Dividing lines indicate splicing within the same gel. Values are represented as means ± s.e.m., *n* per group. Exact *P* values are shown. Statistical differences were determined by a two-sided Student’s *t*-test (normal data; **a**, **d** and **f**–**i**) or a two-sided Mann–Whitney *U* test (non-normal data and non-homogeneous variance; **b**, **c** and **j**). Source data

**Extended Data Fig. 9:**
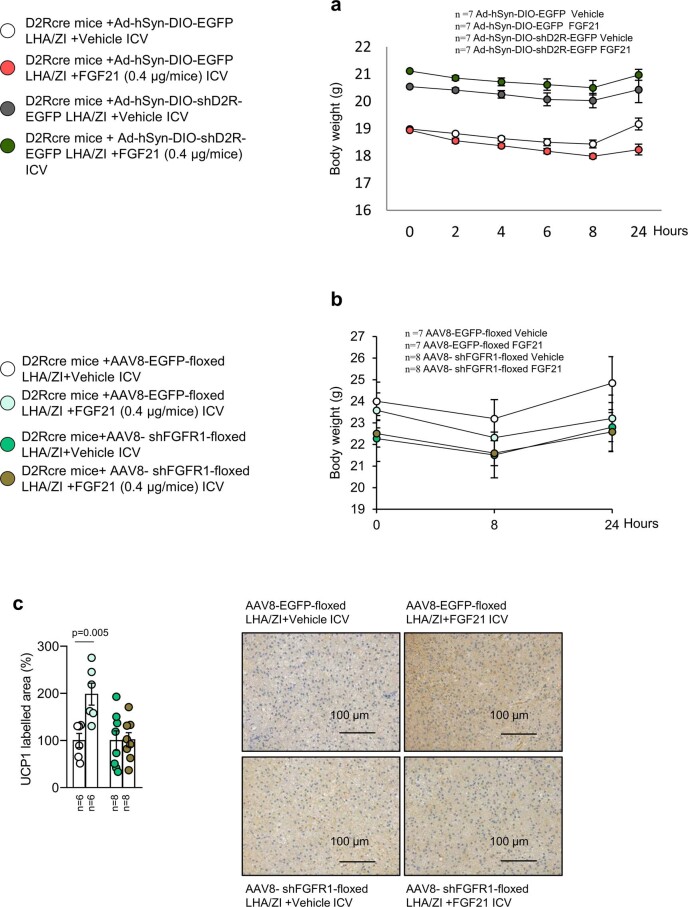
**a**–**c**, FGF21 requires D2R and FGFR1 for its effects. Effect of injecting an adenoviral vector encoding a scrambled RNA (Ad-hSyn-DIO-EGFP) or an shRNA against D2R (Ad-hSyn-DIO-shD2R-EGFP) in a Cre-dependent manner followed by ICV injection of vehicle or FGF21 into D2R-Cre mice on body weight (**a**), effect of injecting an adenoviral vector encoding a scrambled RNA (AAV-EF1A-EGFP-floxed) or an shRNA against FGFR1 (AAV8-EGFP-sh*fgfr1*-floxed) in a Cre-dependent manner, followed by ICV injection of vehicle or FGF21 in D2R-Cre mice on body weight (**b**) and quantification of immunolabeling for UCP1 in BAT (**c**).Values are represented as means ± SEM, n per group indicated in each figure. Exact P values are shown. Statistical differences according to a two-sided Student’s t-test (normal data) (**a**, **b** and **c**). Source data

We next investigated whether FGF21-induced D2R expression was required for the biological actions of FGF21. To test this hypothesis, the AAV encoding sh*D2r* was stereotaxically injected into the LHA/ZI of lean control rats that were subsequently administered ICV FGF21. At the titre and time point studied, *D2r* silencing reversed the effects induced by ICV FGF21 administration on interscapular temperature and protein levels of PGC1α and UCP1 and UCP1 immunolabelling in BAT (Fig. 6a–d). To confirm the requirement of LHA/ZI D2R for the action of FGF21, we then switched to a transgenic mouse model in which D2R-Cre mice were injected into the LHA/ZI with a Cre-dependent AAV encoding either a scrambled RNA (Ad-hSyn-DIO-EGFP) or an shRNA against *D2r* (Ad-hSyn-DIO-sh*D2r*-EGFP). When FGF21 was subsequently ICV administered in these mice, the FGF21-induced expression of c-Fos in LHA/ZI D2R neurons, indicative of their activation, was strongly decreased in the resulting LHA/ZI neuronal population specifically lacking *D2r* (Fig. 6e). Importantly, the FGF21-induced decrease in body weight and the increase in interscapular temperature, UCP1 labelling and PGC1α and UCP1 protein levels in BAT were all prevented in mice lacking *D2r* selectively in the LHA/ZI (Fig. 6f–j and Extended Data Fig. 9a).

**Fig. 7:**
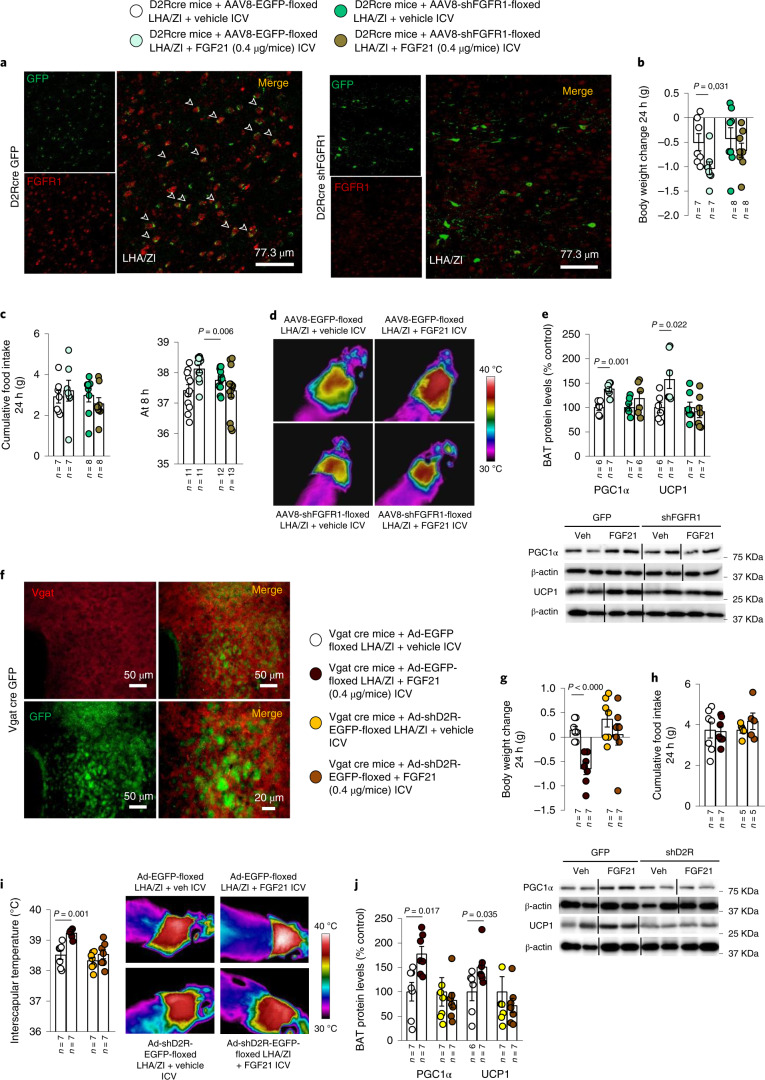
D2R signalling in LHA/ZI GABA neurons is required for FGF21 action. **a**, Photomicrographs showing the colocalization of GFP and FGFR1 in the LHA/ZI of *D2r*-Cre mice. **b**–**e**, Effect of injecting an adenoviral vector encoding a scrambled RNA (AAV-EF1A-EGFP-floxed) or an shRNA against *Fgfr1* (AAV8-EGFP-sh*Fgfr1*-floxed) in a Cre-dependent manner, followed by ICV injection of vehicle or FGF21 in *D2r*-Cre mice on body weight change (**b**); cumulative food intake at 24 h and 8 h (**c**); infrared thermal images and quantification of BAT interscapular temperature (*n* = 11–13; **d**); and BAT protein levels of PGC1α and UCP1 (**e**). **f**, Photomicrographs showing the colocalization of GFP and Vgat in the LHA/ZI of *D2r*-Cre mice. **g**–**j**, Effect of injecting an adenoviral vector encoding a scrambled RNA (Ad-hSyn-DIO-EGFP) or an shRNA against *D2r* (Ad-hSyn-DIO-sh*D2r*-EGFP) in a Cre-dependent manner, followed by ICV injection of vehicle or FGF21 in Vgat-ires-Cre mice on body weight change (**g**), cumulative food intake (**h**); infrared thermal images and quantification of BAT interscapular temperature (**i**); and BAT protein levels of PGC1α and UCP1 (**j**). Protein data were expressed as percentages in relation to control (GFP vehicle) animals. β-actin was used to normalize protein levels. Dividing lines indicate splicing within the same gel. Values are represented as means ± s.e.m., *n* per group. Exact *P* values are shown. Statistical differences were determined by a two-sided Student’s *t*-test (normal data; **b**–**d**, **g** and **h**), or a two-sided Mann–Whitney *U* test (non-normal data and non-homogeneous variance; **e**, **i** and **j**). Source data

Finally, considering that the actions of FGF21 on body weight and BAT thermogenesis appeared to be dependent on *D2r*, we assessed whether FGF21 could act directly on LHA/ZI *D2r* neurons. To this end, the FGF21 receptor (*Fgfr1*) was selectively reduced in LHA/ZI D2R neurons by stereotaxically delivering AAV8-EGFP-floxed or AAV8-sh*Fgfr1*-floxed into the LHA/ZI of *D2r*-Cre mice (Fig. 7a). Similarly to our previous observations, the FGF21-induced decrease in body weight and increase in interscapular temperature, UCP1 labelling and PGC1α and UCP1 protein levels in BAT were abolished in mice with knockdown of FGFR1 in LHA/ZI D2R neurons (Fig. 7b–e and Extended Data Fig. 9b,c), confirming that FGF21 acts directly on these hypothalamic neurons through its receptor, FGFR1.

### D2 receptor in LHA/ZI GABA neurons is required for the FGF21 action

**Extended Data Fig. 10:**
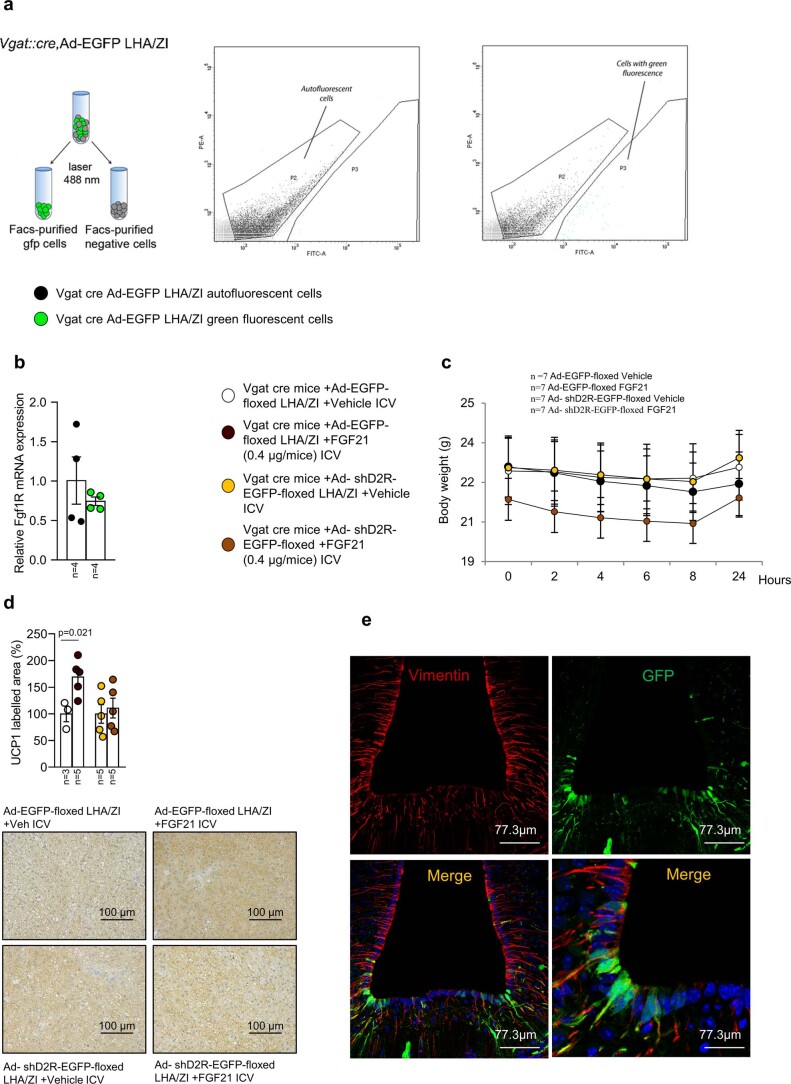
D2R signalling in LHA/ZI GABA neurons is required for FGF21 action. **a**,**b**, Fluorescent activated cell sorting (FACS) to isolate D2R-expressing neurons (GFP-positive) in the LHA/ZI (**a**); FGF21 receptor (FGFR1) mRNA is expressed in isolated D2R cells from the LHA/ZI (**b**). **c**,**d**, Effect of injecting an adenoviral vector encoding a scrambled RNA (Ad-hSyn-DIO-EGFP) or an shRNA against D2R (Ad-hSyn-DIO-sh*D2r*-EGFP) in a Cre-dependent manner, followed by ICV injection of vehicle or FGF21 in Vgat-ires-Cre mice on body weight (**c**) and quantification of immunolabeling for UCP1 in BAT (**d**). **e**, Photomicrograph showing the colocalization of Vimentin and GFP in the lateral ventricle. Source data

Our previous results have revealed that the LHA/ZI neurons required for the thermogenic action of the hypothalamic dopaminergic system are *D2r*-expressing GABAergic neurons^30^. To understand whether the same cell population was also involved in mediating the effects of FGF21, we performed FACS to isolate D2R-expressing GABAergic neurons from the LHA/ZI (Extended Data Fig. 10a). Isolated GABAergic LHA/ZI *D2r* neurons expressed *Fgfr1* mRNA (Extended Data Fig. 10b), suggesting that they could indeed be the effectors of the metabolic actions of FGF21. Accordingly, we knocked down *D2r* specifically in LHA/ZI GABAergic neurons by delivering Ad-EGFP-floxed (controls) or Ad-sh*D2r*-EGFP-floxed into the LHA/ZI of Vgat-Cre mice, as demonstrated by the co-labelling of GFP and Vgat (Fig. 7f). While ICV administration of FGF21 in control mice induced the expected decrease in body weight and an increase in interscapular temperature, UCP1 labelling and PGC1α and UCP1 protein levels in BAT, without altering food intake, these changes were prevented in mice in which D2R was selectively reduced in LHA/ZI GABA neurons (Fig. 7g–j and Extended Data Fig. 10c,d). These findings demonstrate that the metabolic effects of FGF21 are mediated by *D2r*-expressing GABAergic neurons of the LHA/ZI.

### FGF21 is shuttled into the hypothalamus by tanycytes

Finally, we became curious as to the putative route by which peripheral FGF21 could gain access to its target neurons in the hypothalamus. The main gateway for circulating metabolic signals into the hypothalamus is thought to be the median eminence, a circumventricular organ lining the floor of the third ventricle and characterized by capillaries with a fenestrated endothelium. While metabolic signals such as leptin and ghrelin enter the median eminence parenchyma by passive diffusion through the fenestrated capillaries^31,32^ to reach deeper hypothalamic structures, these signals require active transport by specialized hypothalamic glia named tanycytes^32,33^.

**Fig. 8:**
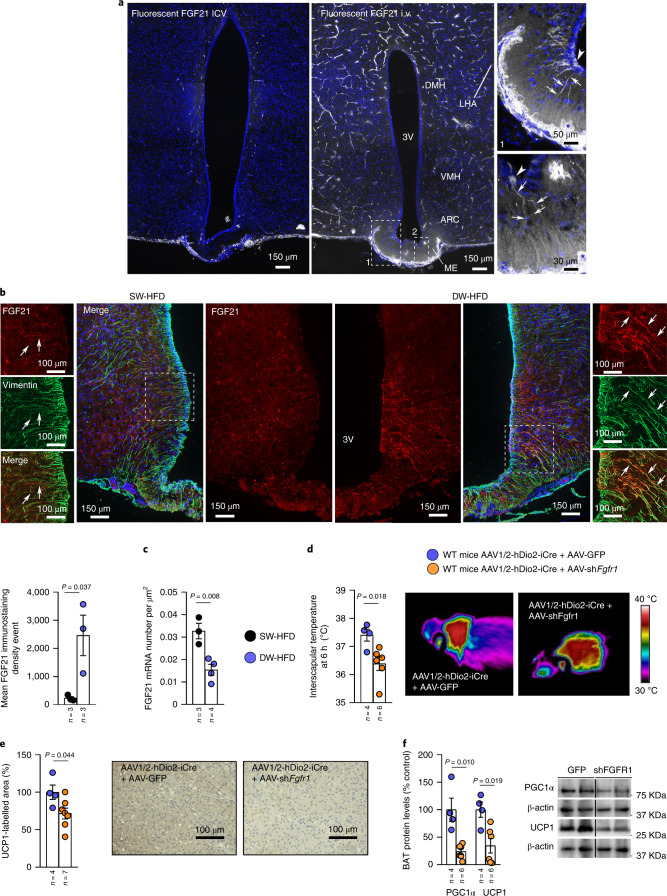
Tanycytic FGFR1 is required for the transport of FGF21 to the hypothalamus. **a**, Representative photomicrographs of the tuberal region of the hypothalamus of a wild-type mouse showing tanycytic processes (arrows) and cell bodies (arrowheads) labelled by fluorescent FGF21 in the median eminence (inset), 1 min after injection into the jugular vein or after the ICV injection. **b**,**c**, Immunoreactivity of FGF21 is increased in tanycytes of SW-HFD and DW-HFD rats (*n* = 3; **b**); mRNA expression of FGF21 in SW-HFD and DW-HFD rats (*n* = 3; **c**). **d**–**f**, Effect of injecting an AAV1/2 into the lateral ventricle expressing Cre recombinase under transcriptional control of the hDio2 promoter (AAV1/2-hDio2-iCre) together with an AAV-GFP-floxed or an shRNA against *Fgfr1* (AAV-GFP-sh*Fgfr1*-floxed) in wild-type (WT) mice on infrared thermal images and quantification of BAT interscapular temperature (*n* = 4–6; **d**); quantification of immunolabelling for UCP1 in BAT (*n* = 4–6; **e**); BAT protein levels of PGC1α and UCP1 (*n* = 4–6; **f**). Protein data were expressed as percentages in relation to control (GFP) animals. β-actin was used to normalize protein levels. Dividing lines indicate splicing within the same gel. Values are represented as means ± s.e.m., *n* per group. Exact *P* values are shown. Statistical differences were determined by a two-sided Student’s *t*-test (normal data; **b**–**e**), or a two-sided Mann–Whitney *U* test (non-normal data and non-homogeneous variance; **f**). Source data

Accordingly, when fluorescently labelled FGF21 was injected through the jugular vein, but not by the ICV route, of control wild-type mice, it was detected in tanycytes of the median eminence (Fig. 8a), suggesting that the same tanycytic shuttle is used to transport hepatic FGF21 into the hypothalamus as is used by other metabolic signals. Interestingly, FGF21 immunoreactivity in DW-HFD rats was significantly increased in tanycytes when compared to SW-HFD rats (Fig. 8b). This increase in FGF21 immunoreactivity in tanycytic processes expressing vimentin, a marker of tanycytes is unlikely due to an upregulation of FGF21 synthesis by the tanycytes themselves because tanycytic *Fgf21* transcripts were seen to be markedly downregulated in DW-HFD rats (Fig. 8c).

Next, to test whether FGFR1-mediated transport of FGF21 through tanycytes may be required for the central regulation of thermogenesis, we inhibited *Fgfr1* expression specifically in tanycytes using AAVs. We gave an ICV injection to wild-type mice with AAV1/2 into the lateral ventricle expressing Cre recombinase under transcriptional control of the human type 2 iodothyronine deiodinase (hDio2) promoter (AAV1/2-hDio2-iCre) together with Cre-dependent AAVs expressing GFP or an shRNA against *Fgfr1*. The efficiency of the approach was demonstrated by assessing the colocalization between GFP and vimentin (Extended Data Fig. 10e). One month after AAV injection, mice were exposed to cold for 6 h and BAT was collected. Mice expressing the *Fgfr1* shRNA in tanycytes showed a lower temperature in the BAT after cold exposure (Fig. 8d), which was consistent with the lower levels of the thermogenic markers PGC1α and UCP1 in BAT (Fig. 8e,f). These results raise the intriguing possibility that FGFR1-mediated FGF21 tanycytic shuttles may be required for the central action of liver FGF21 on BAT activity.

## Discussion

This study reveals that prolonged breastfeeding could counteract the effects of DIO by acting as a long-lasting physiological stimulator of BAT thermogenesis. In adult HFD-fed rats permitted to suckle for an extended period as pups, the activation of the BAT thermogenic programme stimulates energy expenditure, ultimately reducing adiposity, dyslipidaemia and weight gain. The mechanism underlying this protective action involves the increased expression and secretion of hepatic FGF21, which reaches the central nervous system and activates *D2r*-expressing GABAergic neurons located in the LHA/ZI. Taken together, these data imply that by modifying a mother–infant interaction during a specific postnatal temporal window, it is possible to reprogramme hypothalamic circuitries to render the offspring more resistant to DIO in later life.

The potential long-term protective effects of prolonged breastfeeding on energy and metabolic dysfunction in humans are not clear-cut, because the effects of a wide variety of confounding factors affecting both the mother and child (for example, socioeconomic conditions, maternal diet and composition of formula milk) accumulate throughout life^10,34–36^. For this reason, the delayed weaning protocol developed in the present work constitutes an animal model characterized by controlled experimental conditions that minimize confounding factors and enable these long-lasting effects and their underlying mechanisms to be identified and investigated. Our animal model reveals that rats suckled for extended periods are protected against HFD-induced weight gain, glucose intolerance and leptin resistance in adulthood compared with rats suckled for the standard period. This effect is independent of food intake and relies on BAT thermogenesis and the subsequent increase in energy expenditure, indicating that prolonged breastfeeding is a physiological stimulus triggering the function of BAT. These results are in line with a previous report indicating that PPARα, a molecule that induces the thermogenic pathway^37^, is increased in the peripheral blood mononuclear cells of breastfed infants with respect to those fed formula^38^. Notably, when rats subjected to delayed weaning and fed an HFD are exposed to cold, the main physiological activator of thermogenesis, we observed that they were also more efficient at maintaining their body temperature. In addition, prolonged breastfeeding also increases the browning of WAT. The idea that this improved thermogenic action of adipose tissue might be of clinical relevance is supported by evidence showing that BAT activation is inversely correlated with body mass index^39–41^ and that human beige adipocytes seem to have thermogenic capacity^42,43^. However, there are caveats since controversy exists about the role of BAT in human energy metabolism. Some studies indicates that BAT thermogenesis is not clinically relevant in energy expenditure^44^. It was recently suggested that ^18^F-FDG PET/CT, the gold-standard method to quantify total BAT activity in humans^45^, may underestimate the true contribution of BAT to total energy expenditure^44^.

In searching for possible mechanisms underlying the lifelong effects of prolonged breastfeeding, we decided to focus on FGF21, a metabolic hormone that is strongly regulated in mouse neonates. While its levels are very low in fetuses, circulating FGF21 levels rise dramatically after birth and the initiation of suckling; this is associated with the induction of hepatic FGF21 gene expression in response to fatty acids contained in milk^46,47^. Moreover, FGF21 has powerful effects in neonates, including the induction of BAT thermogenesis^48^, and plays a plethora of biological roles in adults such as improving glucose tolerance and enhancing fatty acid oxidation and thermogenic activation^47,49–51^.

In the present model of prolonged breastfeeding, an increase in the levels of FGF21 observed were not due to the transfer of FGF21 from prolonged breastfeeding mothers to the pups. FGF21 protein levels are significantly higher in the livers of pups after delayed weaning and this effect lasts to adulthood, which suggests that the increased hepatic production contributes to the thermogenic activation after prolonged breastfeeding. Supporting the solid effect of the prolonged breastfeeding on thermogenesis still at 4 weeks of age, before the introduction to the HFD, and when the differences in body weight had not yet appeared, the interscapular temperature was already notably increased. These data indicate that changes in BAT thermogenesis precede changes in body weight.

Supporting the role of increased hepatic and circulating FGF21 in response to prolonged breastfeeding in the resistance to DIO, the inhibition of FGF21 in the liver of our animal model reversed the beneficial effects of prolonged breastfeeding on body weight, fat mass and thermogenesis.

It should be taken into account that the use of viral vectors presents several limitations such as rapid clearance and triggering the immune response that diminish the effectiveness of viral therapy (for example, in the review by ref. ^52^). Despite being aware of these limitations, we want to highlight that shRNA viral methods have been extensively used as an efficient tool to investigate the causal role of multiple factors in different organs, and that is what was done here to determine the involvement of key signals such as FGF21 in the liver and D2R in specific hypothalamic neurons. Of note, a control group was always used for each viral vector (lentivirus or AAV sh-scramble) to minimize eventual off-target effects associated with the injection of delivery system. However, we are conscious that this cannot avoid the risks of off-target immune effects exerted by a specific RNA sequence, since in many instances these are often sex-, organ- or tissue-specific effects.

It is well established that FGF21 acts on the central nervous system to induce sympathetic nervous activity, energy expenditure and weight loss^53^. Blood-borne FGF21 has been shown to reach the brain parenchyma at the circumventricular organs, where it extravasates from fenestrated vessels^54^. Here we show that, in the median eminence, extravasating FGF21 is taken up by tanycytes, specialized ependymoglial cells that connect the pituitary portal blood vessels to the third ventricle and are known to actively transport circulating peptide hormones into the CNS in a highly regulated manner^55^. Moreover, the presence of FGF21 receptor in tanycytes is necessary for the central control of BAT activity, which we have shown to convey liver FGF21 action into the hypothalamus. Tanycytes could thus be the major entrance gate for blood-borne FGF21 into the brain, as it appears to be for other key circulating metabolic signals such as leptin^56^, ghrelin^33^ and insulin^57^.

After reaching the brain, FGF21 has been shown to reduce dopamine signalling in brain areas involved in food reward^58^, while maternal HFD feeding is known to reprogramme midbrain dopaminergic circuitry in the midbrain of offspring in mice^59^. However, the possible interaction between FGF21 and dopaminergic circuits controlling body weight has remained unknown until the present. In this regard, our group has recently revealed that the activation of the dopaminergic system in the LHA/ZI of the hypothalamus induces BAT thermogenesis, energy expenditure and weight loss^30^. Our work unifies these various fragmentary findings into a coherent mechanism whereby peripheral FGF21 induced by prolonged breastfeeding protects against the metabolic repercussions of DIO through the activation of hypothalamic dopaminergic pathways. Supporting the relevance of hepatic FGF21 on *D2r* expression in the LHA/ZI, mice lacking FGF21 in the liver, when exposed to cold, displayed lower protein levels of *D2r* in the LHA/ZI together with a decrease in interscapular temperature.

Importantly, FGF21 seems to act directly on a subset of *D2r*-expressing LHA/ZI neurons through its receptor, FGFR1, which forms a complex with β-Klotho to mediate the actions of FGF21 on energy homeostasis^60,61^, including in humans^54^. The LHA and ZI are heterogeneous brain areas containing numerous genetically distinct cell populations. We have previously determined that *D2r* is expressed by both glutamatergic and GABAergic cells, but that only *D2r* expression in GABAergic neurons is responsible for marked effects on body weight when genetically modified^30^. In the present work, we show the thermogenic actions of FGF21 are also mediated specifically by *D2r* expression in GABAergic LHA/ZI neurons. However, FGF21 may act on other hypothalamic areas or neuronal subsets to exert different effects in a specific manner, such as regulating food preference by its action on glutamatergic neurons in the ventromedial hypothalamus^62^.

In summary, this study reveals, firstly, that delayed weaning in rats, which models prolonged breastfeeding in human infants, protects individuals against DIO in adulthood by activating BAT thermogenesis and the browning of WAT, reducing body weight and fat mass, and improving glucose tolerance and leptin sensitivity, without altering food intake. Secondly, these consequences of delayed weaning are the result of an upregulation of the production of hepatic FGF21, which translates into increased circulating levels of FGF21, which in turn enters the brain, likely with the aid of tanycytic shuttles. Thirdly, once in the hypothalamus, FGF21 acts on GABAergic D2R-expressing neurons of the LHA/ZI, which also express the FGF21 receptor FGFR1, to exert its effects on body weight and thermogenesis. In addition to creating new avenues for intervention to improve the metabolic outcome of infants exposed to an HFD, these findings lay the foundations of a better understanding of the mechanisms underlying long-term physiological remodelling by early-life events, with multiple potential health benefits.

## Methods

### Animals and experimental design

Three-month-old female Sprague-Dawley rats fed chow were crossed with 3-month-old males (mean body weight 300 g). The size of the litters was normalized to 12–16 pups (*n* = 6–9 females and 5–8 males). The model of delayed weaning (DW) was developed by extending the period of pups together with their mothers from 21 to 28 d, that is, a weaning at 4 weeks of age. After weaning, only males were used for experiments. Controls were weaned at 3 weeks of age (SW). These groups (DW and SW) were divided at the weaning stage into two subgroups: one fed with a standard CD (Scientific Animal Food & Engineering, 16% protein, 60% carbohydrate and 3% fat) and the second fed with a commercial HFD (Research Diets 12451; 20% protein, 35% carbohydrate and 45% fat, 4.7 kcal g^−1^) until they reached 18 weeks old. In summary, the experimental groups were standard weaning chow diet (SW-CD), delayed weaning chow diet (DW-CD), standard weaning high-fat diet (SW-HFD) and delay weaning high-fat diet (DW-HFD).

The mother rats that breastfed for 21 or 28 d were used. We performed a short time trial, in which SW rats were maintained on a CD until week 4, then put on an HFD until week 5 of age, while DW rats were fed HFD after weaning until 5 weeks of age. The rats were killed at different ages, having the following groups: standard weaning at 3 weeks (SW), delayed weaning at 4 weeks (DW-CD), standard weaning + 1 week of CD (SW-CD), standard weaning + 1 week of CD + 1 week of HFD (SW-CD-HFD), delayed weaning + 1 week of HFD (DW-HFD).

For the generation of male FGF21Alb-KO mice, the FGF21loxP (B6.129S6(SJL)-Fgf21tm1.2Djm/J) line was crossed with (B6.Cg-Tg(Alb-cre)21Mgn/J mice on the C57BL/6J background (Jackson laboratory). B6.Cg-Tg(Alb-cre)21Mgn/J mice (Alb-Cre) were used as controls. In addition, we also used male Drd2-cre mice (C57BL/6J, weight 20–25 g, age 8–10 weeks), Vgat-ires-cre knock-in (C57BL/6J, weight 20–25 g, age 8–10 weeks of age) and their control littermates. Mice were provided water and a standard diet (Scientific Animal Food & Engineering, 16% protein, 60% carbohydrate and 3% fat) ad libitum.

Animals were maintained according to protocols approved by the Animal Care Committee of Santiago de Compostela University (15010/17/007) in accordance with our institutional guidelines and the European Union standards for the care and use of experimental animals. Animals were housed in air-conditioned rooms (22–24 °C), with a controlled light/dark cycle (12 h light, 12 h darkness) and 60% humidity with free access to food and water.

Surgical procedures performed under intraperitoneal (i.p.) anaesthesia of a ketamine–xylazine mixture (ketamine 100 mg per kilogram body weight + xylazine 15 mg per kilogram body weight in rat and ketamine 8 mg per kilogram body weight + xylazine 3 mg per kilogram body weight in mouse). Animals were euthanized by decapitation and trunk blood collected, immediately centrifuged, and plasma stored at −20 °C for the biochemical measurements. The tissues were removed rapidly, frozen on dry ice and kept at −80 °C until analysis.

### Metabolic characterization

We recorded food intake, body weight and body composition (fat mass and lean mass) once a week in rats using an NMR system (Whole-Body Composition Analyzer; EchoMRI). The last week of the experiment, energy expenditure, respiratory quotient and locomotor activity were recorded by calorimetry (LabMaster-multipurpose screening system, TSE Systems)^63,64^. BAT, VAT and GAT were weighed after postmortem.

### Temperature measurements, thermal imaging, cold exposure and thermoneutrality

Body temperature was recorded with a rectal probe connected to a digital thermometer (BAT-12 Microprobe-Thermometer; Physitemp) and the interscapular temperature was measured using a high-resolution infrared camera (E60bx: Compact-Infrared-Thermal Imaging-Camera; FLIR) analysed with a FLIR-Tools-specific software package. Rats were exposed to cold (4 °C) for 6 h and then moved to a thermoneutral environment (30 °C with relative humidity of 45–52%) to eliminate the extra metabolism needed to defend the body temperature at lower temperatures^30^.

### PET acquisition protocols

Sprague-Dawley rats were analysed with a PET/CT Preclinical Imaging System (Bruker Biospin). Approximately 12.32 ± 0.47 MBq of ^18^F-FDG radiotracer was injected in the tail vein under anaesthesia (2–2.5% of isoflurane). PET radiotracer ^18^F-FDG was used to assess cell glucose metabolism, formed by a glucose molecule with the positron-emitting radionuclide ^18^F substituted for the normal hydroxyl group at the C-2 position. The animals woke up a few minutes later and were kept at rest for 35 min or kept at 4 °C for 28 min with access to food and water. After that, the animals were again anaesthetized, and PET static acquisitions performed at 45 min after injection (10 min of scan). PET images were reconstructed using the maximum likelihood expectation maximization algorithm with 12 iterations and image pixel size of 0.5 × 0.5 × 0.5 mm^3^. The field of view of PET scan was centred in the cervical region of the animal.

Images were analysed with AMIDE software (https://sourceforge.net/projects/amide/). Quantitative analysis was done by spherical regions of interest with dimensions of 6 × 6 × 6 mm delineated on the iBAT of each animal to capture the maximum ^18^F-FDG uptake value in the iBAT. Standardized uptake value (SUVmax; proportional to the glucose metabolism) was calculated as the maximum ^18^F-FDG uptake value normalized by the injected activity and the body weight of the animal. The injected ^18^F-FDG activity was estimated by subtracting the extravasated activity in the tail.

### Glucose and insulin tolerance tests

A glucose tolerance test and an insulin tolerance test were performed by i.p. injection of glucose (2 g per kg body weight) or insulin (0.50 U per kg body weight) after overnight fasting. For glucose measurement, a small cut was made near the end of the rat tail, and the drops of blood collected directly on the test strip placed in the blood glucometer (Glucocard G + meter, A. Menarini diagnosis). Blood samples were collected immediately before and 15, 30, 60, 90 and 120 min after glucose administration^65^.

### Breast milk extraction

After weaning, the mothers were housed for approximately 4 h and subsequently sedated with isofluorane and 5 IU of oxytocin (Syntocinon 10 IU per ml, Alfasigma) was administered subcutaneously. Next, the breasts were massaged, and milk obtained directly in a tube (for a maximum of 1 h) and centrifuged for 10 min at 800*g* at 4 °C, leaving us with an intermediate phase.

### Tail vein injection

Rats were held in a specific restrainer for tail vein injections (Tailveiner; TV-150, Bioseb). With a 27G 3/8-inch (0.40 mm × 10 mm) syringe, rats were injected with 500 μl of lentiviral vectors diluted in saline. For the downregulation of FGF21 specifically in the liver, we used shLuciferase or shRNA knockdown FGF21 lentiviral vectors (1 × 10^9^ plaque-forming units (PFUs) per ml)^66^.

The specific shRNAs for knockdown of Fgf21 transcripts in rat and shLuciferase shRNA were designed, synthesized and subcloned into lentiviral pLKO.1 plasmid^67^ (plasmid no. 8453; Addgene, RRID: 8453)^25^. ShRNA constructs were confirmed by Sanger sequencing and knockdown efficiency validated by RT–qPCR. The target sequences of the shRNAs used were sh-scrambled CCTAAGGTTAAGTCGCCCTCG; sh-Fgf21_2 TCTCTATGGATCGCCTCACTT. Lentiviral pLKO.shRNA-expressing vectors were co-transfected with psPAX2 and pMD2.G packaging vectors in HEK293T cells (American Type Culture Collection, CRL-3216) were maintained in high-glucose DMEM with 10% FBS, 2 mM l-glutamine and 1% penicillin–streptomycin. Cells were plated at density of 8 × 10^6^ cells per 150-mm dish, 8 million HEK293T cells were seeded onto 150-mm dishes and transfected 24 h later with 20 µg of the corresponding pLKO.shRNA plasmid and 10 µg of psPAX2 and pMD2.G packaging plasmids using polyethylenimine (Sigma-Aldrich, 408727). Twenty-four hours later, medium was changed, and virus-containing supernatants were collected 48 and 72 h after transfection and concentrated using a centrifugal filter (0.22-µm pore size; Amicon, UFC903024).^25,67^.

### Jugular vein injection of fluorescent FGF21

Five nanomoles of fluorescent recombinant FGF21 (Cisbio Bioassays)^54^ was injected into the jugular vein of anaesthetized adult mice and, 1 min later, the animals were killed. Brains were immersion-fixed in 4% paraformaldehyde (0.1 M PBS pH 7.4) for 2 h and placed in the same fixative + 20% sucrose overnight at 4 °C. Brains were frozen in liquid-nitrogen-cooled isopentane and 30-µm-thick cryostat sections were collected on superfrost glass slides. Images were acquired with an Axio Imager Z2 microscope (Zeiss). Fluorescent leptin was imaged by using a 660-nm beam splitter (excitation wavelength set at 625/655 nm and an emission wavelength set at 665/715 nm).

### Intracerebroventricular treatment

In rats, anaesthesia was administered by i.p. injection (ketamine–xylazine mixture), and a cannula implanted by stereotaxic surgery in the hypothalamic lateral ventricle (coordinates: 1.3 mm posterior to bregma, 1.9 mm lateral to the midsagittal suture, and a depth of 3.5 mm; and in mice: 0.6 mm posterior to bregma, 1.2 mm lateral to the midsagittal suture and a depth of 2 mm)^20^. Animals were individually stabled 4 d before the experiment. ICV vehicle (saline), leptin (3 μg per rat, provided by Albert Parlow) or FGF21 (recombinant human FGF21 at 0.4 μg per animal; ProSpec) was administered.

### Stereotaxic microinjections in specific hypothalamic nuclei and lateral ventricle of adenoviral expression vectors

The animals, after anaesthesia with ketamine–xylazine, were disposed in a stereotaxic frame (David Kopf Instruments). Localization of the LHA/ZI in rats for the stereotaxic injection coordinates: anterior to the bregma (AP), −2.85 mm; lateral to the sagittal suture (L), ± 2 mm; and ventral from the surface of the skull (DV), −8.1 mm; and in mice were AP, −1.3 mm; L, ±1.1 mm; DV, −5.2 mm. Mice lateral ventricle coordinates were AP, −0.3 mm; L, ±1 mm; DV, −2.5 mm. Animals were i.p. treated with an analgesic (acetylsalicylic acid, Bayer; 150 mg per kilogram body weight). Adenoviral vector D2R knockdown (3.5 × 10^10^ PFUs per ml) or vector controls (3.5 × 10^10^ PFUs per ml) were administered in rats.

The modification of D2R expression was performed using Ad-hSyn-DIO-shD2R-EGFP (1.0 × 10^10^ PFUs per ml) and Ad-hSyn-DIO-EGFP (1.0 × 10^10^ PFUs per ml; Vector Builder) under cell-specific cre promoters^30,68^.

For the specific modulation of FGFR1 expression, we used AAV-shFGFR1-EGFP-floxed (1.0 × 10^13^ PFUs per ml) and AAV-EGFP-floxed (2.48 × 10^13^ PFUs per ml; Vector Builder) under the cell-specific cre driver line. To modify the FGFR1 specifically in tanycytes, we injected AAV1/2 into the lateral ventricle expressing Cre recombinase under transcriptional control of the hDio2 promoter (AAV1/2-hDio2-iCre) together with an AAV-GFP-floxed or AAV-GFP-sh*Fgfr1*-floxed.

### Dissection of brain areas

Brains were removed and immediately frozen and stored at −80 °C until processing. Then, they were placed in a brain matrix with the ventral surface on top under a dissecting microscope. LHA/ZI and MBH were excised from the whole hypothalamus cutting between the rostral and caudal limits of the median eminence parallel to the base of the hypothalamus and 1 mm to each lateral side of the median eminence for LHA/ZI and 0.5 mm for MBH. The depth of each section isolated was around 1 mm thick in mice and 3 mm thick in rat brain^20^.

### Histomorphology and immunohistochemistry

Tissues were fixed in 10% paraformaldehyde for inclusion in paraffin. BAT and SAT samples were cut and mounted in a section (3 μm) and stained (haematoxylin and eosin alcoholic (BioOptica) procedure)^64^. Frozen sections of liver were cut (8 µm) with a cryostat and stained in filtered Oil Red O (10 min). Sections were washed in distilled water, counterstained with Mayer’s haematoxylin (3 min), mounted in aqueous mounting medium (glycerine jelly) and observed and photographed with a Zeiss AXIO microscope digitizing the images with a coupled camera (AxioCam MRC) and quantified with ImageJ software (RRID: SCR_003070).

### Immunofluorescence

Mice brains were fixed by perfusion and immersion in 10% buffered formalin (24 h). Brain pieces were cut (40 μm) using freezing microtome Leica CM1850 UV cryostat. Sections were washed three times in 0.1 M TBS (10 min) each and incubated in blocking solution (2% donkey serum + 0.3% Triton X-100) in 0.1 M TBS (60 min). Then, sections were incubated in rabbit anti-c-Fos or chicken anti-GFP, rabbit anti-VGat, rabbit anti-FGFR1, vimentin or DAPI in blocking solution for 24 h at 4 °C. After that, sections were rinsed with 0.1 M TBS three times (10 min) each and incubated in the secondary antibody: Cy3 donkey anti-rabbit and goat anti-chicken Alexa 488 (60 min at room temperature). Sections were then washed and coverslipped with Fluorogel. Confocal images were collected with Leica A0B5-SP5 microscope.

### Levels of plasma metabolites and hormones

Plasma levels of triglycerides, total cholesterol and free fatty acids were determined by specific commercial kits (triglycerides and cholesterol kit from Spinreact; free fatty acids kit from Wako), based on a colorimetric enzymatic reaction (Epoch 2 microplate reader, BioTek Instruments).

Circulating levels of leptin and FGF21 were determined by enzymatic immunoassay (ELISA) using commercial kits. For leptin, the sensitivity limit of the assay was 0.08 ng ml^−1^ (Millipore, EZRL-83K, RRID: AB_2307316), and, for FGF21, the sensitivity limit was 3.81 pg ml^−1^ (R and D Systems, MF2100, RRID: AB_2783730).

### FACS sorting

The tuberal region of the hypothalamus from Vgat-cre mice injected with EGFP adenovirus in LHA/ZI was microdissected and then enzymatically dissociated with Papain Dissociation System (Worthington) to single-cell suspensions^69^. FACS was performed using an ARIA SORP Cell Sorter Cytometer device (BD Bioscience). The sort selection was based on measurements of EGFP fluorescence (excitation: 488 nm; 50 mW; detection: EGFP bandpass, 525/30 nm; autofluorescence bandpass, 695/40 nm) comparing cell suspensions from non-infected (that is, cortex) and infected (that is, the hypothalamus) brain sites (Fig. 5a). For each animal, 150 to 200 EGFP-positive cells were sorted directly into 10 μl of extraction buffer: 0.1% Triton X-100 (Sigma-Aldrich) and 0.4 units per μl RNaseOUT (Life technologies). RNAs collected from FACS-sorted EGFP-negative and positive cells were reversed transcribed using High-Capacity Reverse Transcription (Life Technologies) and the linear preamplification stage was carried out with the TaqMan PreAmp Master Mix Kit Protocol (4366128, Applied Biosystems). Real-time PCR was performed on the Applied Biosystems 7900HT Fast Real-Time PCR system using exon-boundary-specific TaqMan Gene Expression Assays (Applied Biosystem): FGFR1 (*Fgfr1-*Mm00438930_m1). The control housekeeping genes used were R18S (*r18S*-Mm03928990_g1) and Actin (*Actb*-Mm00607939_s1).

### Fluorescence in situ hybridization combined to immunofluorescence

FISH was performed on 40-µm brain free-floating coronal sections of the MBH of rats perfused with 10% formalin with the RNAscope Multiplex Fluorescent Kit v2 to detect *Fgf21* (170580, NM_130752.1, target regions 9–620) mRNAs and coupled to an immunohistochemistry for the rabbit monoclonal antibody anti-FGF21 and the chicken polyclonal antibody anti-vimentin. FISH was performed according to the manufacturer’s instructions, with some modification for free-floating sections described by Grabinski et al.^70^. Hybridization with a probe against the *Bacillus subtilis* dihydrodipicolinate reductase (*dapB*) gene (320871) was the negative control. For microscopy, image acquisition was performed using an inverted confocal microscope (LSM 710, Zeiss). Excitation wavelengths of 493/562 nm, 568/643 nm and 640/740 nm were selected to image Alexa 488 and Alexa 647 secondary antibodies and Cyanine 3. An ultraviolet laser (wavelength of 355 nm) was used to image DAPI. *Z*-stack images were acquired with a W Plan-APOCHROMAT ×20 objective (NA of 0.5, zoom of 1.0).

### Quantitative analyses

#### FGF21 immunostaining

Using the ImageJ analysis software, the maximal intensity projection images were binarized to compensate for differences in fluorescence intensity. Images were skeletonized so that each tanycyte process was 1-pixel thick. Mean area and integrated density were calculated for each image, proportional to the total surface of the staining and to the complexity of the process’s organization.

#### FGF21 in situ hybridization

The quantification has been performed on the ependymal layer bordering the third ventricle in the median eminence and the arcuate nucleus, corresponding to the localization of tanycyte nuclei. Using Zen imaging software, the area of quantification was determined and the total number of dots per µm^2^ calculated.

### Western blot analysis

Tissues were homogenized with TissueLyser II (Qiagen) in cold RIPA buffer (200 mM Tris–HCl (pH 7.4), 130 mM sodium chloride, 10% (vol/vol) glycerol, 0.1% (vol/vol) SDS, 1% (vol/vol) Triton X-100 and 10 mM magnesium chloride) with antiproteases and antiphosphatases (Sigma-Aldrich). The lysates were centrifuged (30 min, 18,000*g*, 4 °C). BAT, SAT, MBH, liver and LHA total protein lysates were subjected to SDS–PAGE, electrotransferred onto a PVDF membrane and probed with the following antibodies: pSTAT3, STAT3, pPI3K, PI3K, pAKT, AKT, pERK, ERK, PPARγ, PGC1α, UCP1, pHSL, HSL, FGF21, D2R and orexin A and B. Protein levels were normalized with β-actin for each sample. Membranes were incubated with 5% BSA blocking buffer. Detection of proteins was performed using horseradish-peroxidase-conjugated secondary antibodies.

Specific antigen–antibody binding was visualized with chemiluminescence method according to the manufacturer’s instructions (Pierce ECL Western Blotting Substrate, Thermo Scientific), manual development of X-ray films or the ChemiDoc Imaging System. ImageJ (RRID: SCR_003070) was used to quantify western blot analysis.

### Statistical analysis

Results are given as means ± s.e.m. The number of animals used in each study is listed in the figure legends. Statistical analyses were performed using IBM SPSS Statistics (RRID: SCR_019096). To evaluate whether the data follow a normal distribution and variances are homogeneous, the Kolmogorov–Smirnov test and the Levene test, respectively, were performed. The comparison between groups was performed using ANOVA (normal data and homogeneity of variances) followed by Tukey’s post hoc multiple-comparison test or a two-sided Student’s *t*-test (normal data), ANCOVA and two-sided Mann–Whitney *U* test (non-normal data and non-homogeneous variance). The level of statistical significance was set at a *P* value less than 0.05.

### Reporting summary

Further information on research design is available in the Nature Research Reporting Summary linked to this article.

## Supplementary information

Supplementary Information Supplementary Table 1

Reporting Summary

## Data Availability

Source data are provided with this paper.

## Source data

Source Data Fig. 1 Statistical source data.
Source Data Fig. 1 Unprocessed western blots.
Source Data Fig. 2 Statistical source data.
Source Data Fig. 3 Statistical source data.
Source Data Fig. 3 Unprocessed western blots.
Source Data Fig. 4 Statistical source data.
Source Data Fig. 4 Unprocessed western blots.
Source Data Fig. 5 Statistical source data.
Source Data Fig. 5 Unprocessed western blots.
Source Data Fig. 6 Statistical source data.
Source Data Fig. 6 Unprocessed western blots.
Source Data Fig. 7 Statistical source data.
Source Data Fig. 7 Unprocessed western blots.
Source Data Fig. 8 Statistical source data.
Source Data Fig. 8 Unprocessed western blots.
Source Data Extended Data Fig. 1 Statistical source data.
Source Data Extended Data Fig. 2 Statistical source data.
Source Data Extended Data Fig. 3 Statistical source data.
Source Data Extended Data Fig. 3 Unprocessed western blots.
Source Data Extended Data Fig. 4 Statistical source data.
Source Data Extended Data Fig. 4 Unprocessed western blots.
Source Data Extended Data Fig. 5 Statistical source data.
Source Data Extended Data Fig. 5 Unprocessed western blots.
Source Data Extended Data Fig. 6 Statistical source data.
Source Data Extended Data Fig. 6 Unprocessed western blots.
Source Data Extended Data Fig. 7 Statistical source data.
Source Data Extended Data Fig. 7 Unprocessed western blots.
Source Data Extended Data Fig. 8 Statistical source data.
Source Data Extended Data Fig. 9 Statistical source data.
Source Data Extended Data Fig. 10 Statistical source data.
