# Supplementary material for: Prolonged breastfeeding protects from obesity by hypothalamic action of hepatic FGF21
Source: Nat Metab. 2022 Jul 25;4(7):901–17. doi: 10.1038/s42255-022-00602-z (PMC9314260; doi:10.1038/s42255-022-00602-z)
Supplement: Supplementary file 1 — Supplementary Table 1 [file 42255_2022_602_MOESM1_ESM.pdf]

nature portfolio

https://doi.org/10.1038/s42255-022-00602-z

Supplementary information

Prolonged breastfeeding protects from obesity by hypothalamic action of hepatic FGF21

In the format provided by the authors and unedited

Supplementary table 1. Antibodies used for western blot.

Antibody	Dilution	Vendor	Catalog number	RRID
Anti-pSTAT3	1:1000	Cell Signaling Technology	9134	AB_331589
Anti-STAT3	1:1000	Cell Signaling Technology	4904	AB_331269
Anti-pPI3K	1:1000	Cell Signaling Technology	4228	AB_659940
Anti-PI3K	1:1000	Cell Signaling Technology	4292	AB_329869
Anti-pAKT	1:1000	Cell Signaling Technology	9271	AB_329825
Anti-AKT	1:1000	Cell Signaling Technology	9272	AB_329827
Anti-pERK	1:1000	Cell Signaling Technology	4370	AB_2315112
Anti-ERK	1:1000	Cell Signaling Technology	9102	AB_330744
Anti-PPARγ	1:1000	Abcam	ab27649	AB_777390
PGC1α	1:1000	Abcam	ab54481	AB_881987
UCP1	1:1000	Abcam	ab10983	AB_2241462
pHSL	1:1000	Cell Signaling Technology	4126	AB_490997
HSL	1:1000	Abcam	ab45422	AB_2135367
FGF21	1:1000	Abcam	ab64857	AB_2104485
D2R	1:1000	Abcam	ab85367	AB_10674739
β-ACTIN	1:5000	Sigma-Aldrich	A5316	AB_476743

OX A/B	1:1000	Santa Cruz Biotechnology	sc-28935	AB_784981
Goat Anti-rabbit	1:5000	Jackson ImmunoResearch Labs	111-035-003	AB_2313567
Goat Anti-mouse	1:10000	Jackson ImmunoResearch Labs	115-035-003	AB_10015289
