# Supplementary material for: Prolonged breastfeeding protects from obesity by hypothalamic action of hepatic FGF21
Source: Nat Metab. 2022 Jul 25;4(7):901–17. doi: 10.1038/s42255-022-00602-z (PMC9314260; doi:10.1038/s42255-022-00602-z)
Supplement: Supplementary file 2 — Reporting Summary [file 42255_2022_602_MOESM2_ESM.pdf]

nature research

Corresponding author(s): Rubén Nogueiras

Last updated by author(s): Jun 7, 2022

Reporting Summary

Nature Research wishes to improve the reproducibility of the work that we publish. This form provides structure for consistency and transparency in reporting. For further information on Nature Research policies, see our Editorial Policies and the Editorial Policy Checklist.

Statistics

For all statistical analyses, confirm that the following items are present in the figure legend, table legend, main text, or Methods section.

n/a	Confirmed	
☐	☒	The exact sample size (*n*) for each experimental group/condition, given as a discrete number and unit of measurement
☐	☒	A statement on whether measurements were taken from distinct samples or whether the same sample was measured repeatedly
☐	☒	The statistical test(s) used AND whether they are one- or two-sided<br>*Only common tests should be described solely by name; describe more complex techniques in the Methods section.*
☐	☒	A description of all covariates tested
☐	☒	A description of any assumptions or corrections, such as tests of normality and adjustment for multiple comparisons
☐	☒	A full description of the statistical parameters including central tendency (e.g. means) or other basic estimates (e.g. regression coefficient) AND variation (e.g. standard deviation) or associated estimates of uncertainty (e.g. confidence intervals)
☐	☒	For null hypothesis testing, the test statistic (e.g. *F*, *t*, *r*) with confidence intervals, effect sizes, degrees of freedom and *P* value noted<br>*Give P values as exact values whenever suitable.*
☒	☐	For Bayesian analysis, information on the choice of priors and Markov chain Monte Carlo settings
☒	☐	For hierarchical and complex designs, identification of the appropriate level for tests and full reporting of outcomes
☒	☐	Estimates of effect sizes (e.g. Cohen's *d*, Pearson's *r*), indicating how they were calculated

Our web collection on statistics for biologists contains articles on many of the points above.

Software and code

Policy information about availability of computer code

Data collection	Image-J software, GraphPad Prism 8, SPSS/Windows version 15.0 and FLIR-Tools software. All this is also mentioned in the Methods section.
Data analysis	Image-J software, AMIDE software, Zen imaging software, GraphPad Prism 8, and SPSS/Windows version 15.0. All this is also mentioned in the Methods section.

For manuscripts utilizing custom algorithms or software that are central to the research but not yet described in published literature, software must be made available to editors and reviewers. We strongly encourage code deposition in a community repository (e.g. GitHub). See the Nature Research guidelines for submitting code & software for further information.

Data

Policy information about availability of data

All manuscripts must include a data availability statement. This statement should provide the following information, where applicable:

- Accession codes, unique identifiers, or web links for publicly available datasets
- A list of figures that have associated raw data
- A description of any restrictions on data availability

Source data are available as supplementary material

Field-specific reporting

Life sciences study design

All studies must disclose on these points even when the disclosure is negative.

Sample size	For animal experiments, sample size was chosen based on similar previous studies of our group and on the basis of literature documentation of similar wellcharacterized experiments. We try to use the fewest number of animals to achieve statistical significance without compromising the outcomes. The sample size are provided in figures. Analysis section in Methods.
Data exclusions	Samples of animals were excluded whether their values were outside the 2SD range according to proper statistical analyses. 1 rat of the delayed weaning model and 1 D2Rcre mouse injected with AAV-shFGFR1 were excluded.
Replication	Experimental technical duplicates were included in all measurements in this study, ensuring the reproducibility of the data.
Randomization	Before start an experiment, all animals groups were made with set of animals of the same sex, age and similar body weight.
Blinding	For practical reasons, the investigators were not blinded to allocation during in vivo experiments. Animal samples received a correlative number independent of their genotype, ensuring the blindness of data collection analyses.

Reporting for specific materials, systems and methods

We require information from authors about some types of materials, experimental systems and methods used in many studies. Here, indicate whether each material, system or method listed is relevant to your study. If you are not sure if a list item applies to your research, read the appropriate section before selecting a response.

Materials & experimental systems

n/a	Involved in the study	
☐	☒	Antibodies
☐	☒	Eukaryotic cell lines
☒	☐	Palaeontology and archaeology
☐	☒	Animals and other organisms
☒	☐	Human research participants
☒	☐	Clinical data
☒	☐	Dual use research of concern

Methods

n/a	Involved in the study	
☒	☐	ChIP-seq
☐	☒	Flow cytometry
☒	☐	MRI-based neuroimaging

Antibodies

Antibodies used

The following antibodies were used:

Anti-pSTAT3 (1:1000) Cell Signaling Technology Cat# 9134, RRID:AB_331589
Anti-STAT3 (1:1000) Cell Signaling Technology Cat# 4904, RRID:AB_331269
Anti-pPI3K (1:1000) Cell Signaling Technology Cat# 4228, RRID:AB_659940
Anti-PI3K (1:1000) Cell Signaling Technology Cat# 4292, RRID:AB_329869
Anti-pAKT (1:1000) Cell Signaling Technology Cat# 9271, RRID:AB_329825
Anti-AKT (1:1000) Cell Signaling Technology Cat# 9272, RRID:AB_329827
Anti-pERK (1:1000) Cell Signaling Technology Cat# 4370, RRID:AB_2315112
Anti-ERK (1:1000) Cell Signaling Technology Cat# 9102, RRID:AB_330744
Anti-PPARγ (1:1000) Abcam Cat# ab27649, RRID:AB_777390
PGC1α (1:1000) Abcam Cat# ab54481, RRID:AB_881987
UCP1 (1:1000) Abcam Cat# ab10983, RRID:AB_2241462
pHSL (1:1000) Cell Signaling Technology Cat# 4126, RRID:AB_490997
HSL (1:1000) Abcam Cat# ab45422, RRID:AB_2135367
FGF21 (1:1000) Abcam Cat# ab64857, RRID:AB_2104485
D2R (1:1000) Abcam Cat# ab85367, RRID:AB_10674739
β-ACTIN (1:1000) Sigma-Aldrich Cat# A5316, RRID:AB_476743
OX A/B (1:1000) Santa Cruz Biotechnology Cat# sc-28935, RRID:AB_784981
Goat Anti-rabbit (1:5000) Jackson ImmunoResearch Labs Cat# 111-035-003, RRID:AB_2313567
Goat Anti-mouse (1:10000) Jackson ImmunoResearch Labs Cat# 115-035-003, RRID:AB_10015289
Rabbit anti-cFos (1/200) Santa Cruz, Cat# sc-52, RRID:AB_2106783
Chicken anti-GFP (1/1000) Invitrogen A10262 Cat# 10524234, RRID:AB_2534023
Rabbit anti-VGat (1/1,000) Synaptic Systems, Cat# 131 013, RRID:AB_2189938
Rabbit anti-FGFR1 (1/200) Abcam Cat# ab10646, RRID:AB_297367
Cy3 donkey anti-rabbit (1:1000) Jackson ImmunoResearch Labs Cat#711-165-152, RRID:AB_2307443
Goat anti-chicken Alexa 488 (1:1000) abcam Cat# ab150169, RRID:AB_2636803

	Vimentin (1/1000) Millipore Cat# AB5733, RRID:AB_11212377
Validation	All antibodies used in the paper have been validated by the manufacturer and by references:<br><br>Anti-pSTAT3: This antibody has been validated using SimpleChIP® Enzymatic Chromatin IP Kits.<br>Anti-STAT3: This antibody has been validated using SimpleChIP® Enzymatic Chromatin IP Kits.<br>Anti-pPI3K:PMID: 25743370, PMID: 32150791, PMID: 31131539<br>Anti-PI3K: PMID: 25743370, PMID: 32150791, PMID: 31131539<br>Anti-pAKT: PMID: 31579887, PMID: 30853298, PMID: 31746399<br>Anti-AKT: PMID: 31579887, PMID: 29021135, PMID: 31311969<br>Anti-pERK:PMID: 28049723, PMID: 35317201, PMID: 35203351<br>Anti-ERK: PMID: 28049723, PMID: 35040015, PMID: 35203351<br>Anti-PPARγ: PMID: 33939165, PMID: 32184391, PMID: 26171158<br>PGC1α:PMID: 33239403, PMID: 33705351, PMID: 33374300<br>UCP1: PMID: 33334822, PMID: 33171307, PMID: 33166186<br>pHSL: PMID: 28799896, PMID: 34508100, PMID: 34006859<br>HSL:PMID: 34085745, PMID: 28683288, PMID: 28053001<br>FGF21: PMID: 31579887, PMID: 34850960, PMID: 24062250<br>D2R: PMID: 31579887, PMID: 33933677, PMID: 32060266<br>β-ACTIN: The isotype is determined using Sigma ImmunoType Kit (Product Code ISO-1) and by a double diffusion immunoassay using Mouse Monoclonal Antibody Isotyping Reagents (Product Code ISO-2).<br>OX A/B: PMID: 9742163, PMID: 9491897,PMID: 9419374<br>Goat Anti-rabbit: PMID 26961074, PMID: 25743370, PMID: 30266914<br>Goat Anti-mouse:PMID 26961074, PMID: 25743370, PMID: 30266914<br>Rabbit anti-cFos: PMID: 31579887, PMID:35078818, PMID:33169700<br>Chicken anti-GFP: PMID: 34324439, PMID: 33744652, PMID: 32451441<br>Rabbit anti-VGat: PMID: 31579887, PMID: 34845591, PMID: 32265258<br>Rabbit anti-FGFR1: PMID:33810560, PMID: 33495338, PMID: 33217323<br>Cy3 donkey anti-rabbit: PMID: 33744652, PMID: 28487659, PMID: 28799896<br>Goat anti-chicken Alexa 488: PMID: 33744652, PMID: 33436964, PMID: 33666172<br>Vimentin: PMID: 34341568, PMID: 26337286, PMID: 25721933

Eukaryotic cell lines

Policy information about cell lines

Cell line source(s)	HEK293T cells were purchased from ATCC, # CRL-3216
Authentication	The cell line used was not authenticated in the laboratory
Mycoplasma contamination	The cell line tested negative for mycoplasma
Commonly misidentified lines<br>(See ICLAC register)	N/A

Animals and other organisms

Policy information about studies involving animals; ARRIVE guidelines recommended for reporting animal research

Laboratory animals	3-month-old male and female Sprague-Dawley rats (standard laboratory diet (CD): 200-250g and high fat diet (HFD) for 12 weeks: 450-700g. Male WT and Drd2-cre:ribotag mice (weight 20–25 g, age 8–10 weeks old) and vgat-ires-cre knock-in (C57BL/6J) from Jackson Laboratory (weight 20-25g, age 8-10 weeks old) also were used for the experiments. For the generation of FGF21Alb-KO male mice, the FGF21loxP (B6.129S6(SJL)-Fgf21tm1.2Djm/J) line was crossed with (B6.Cg-Tg(Alb-cre)21Mgn/J mice on the C57BL/6J background (Jackson laboratory). Animals were housed in air-conditioned rooms (22-24ºC), controlled light/dark cycle (12 hours light, 12 hours darkness) and humidity (60%) with free access to food and water. All this information about animal species, sex, strain, provider, etc are mentioned in the Material and Methods section.
Wild animals	This study did not involve wild animals.
Field-collected samples	This study did not involve field collected samples.
Ethics oversight	All experiments and procedures involved in this study were reviewed and approved by the Ethics Committee of the University of Santiago de Compostela, in accordance with European Union normative for the use of experimental animals.

Note that full information on the approval of the study protocol must also be provided in the manuscript.

Flow Cytometry

Plots

Confirm that:

- [x] The axis labels state the marker and fluorochrome used (e.g. CD4-FITC).
- [x] The axis scales are clearly visible. Include numbers along axes only for bottom left plot of group (a 'group' is an analysis of identical markers).
- [x] All plots are contour plots with outliers or pseudocolor plots.
- [x] A numerical value for number of cells or percentage (with statistics) is provided.

Methodology

Sample preparation	The tuberal region of the hypothalamus of Vgat-cre + Ad-EGFP LHA/ZI mice were microdissected and enzymativcally dissociated using Papain Dissociation System (Worthington, Lakewood, NJ) to obtain single cell suspensions as described previously
Instrument	FACS was performed using an EPICS ALTRA Cell Sorter Cytometer device (BD Bioscience).
Software	BD FACSuite Software
Cell population abundance	The sort decision was based on measurements of EGFP fluorescence (excitation: 488 nm; 50 mW; detection: EGFP bandpass 530/30 nm, autofluorescence bandpass 695/40 nm) by comparing cell suspensions from non-infected brain sites and infected brain sites.
Gating strategy	The sort decision was based on measurements of EGFP fluorescence (excitation: 488 nm; 50 mW; detection: EGFP bandpass 530/30 nm, autofluorescence bandpass 695/40 nm) by comparing cell suspensions from non-infected brain sites and infected brain sites.

- [x] Tick this box to confirm that a figure exemplifying the gating strategy is provided in the Supplementary Information.
