# Supplementary figures and images for: Prolonged breastfeeding protects from obesity by hypothalamic action of hepatic FGF21
Source: Nat Metab. 2022 Jul 25;4(7):901–17. doi: 10.1038/s42255-022-00602-z (PMC9314260; doi:10.1038/s42255-022-00602-z)

### Supplementary Figure 1

### Uncropped blots Figure 1k

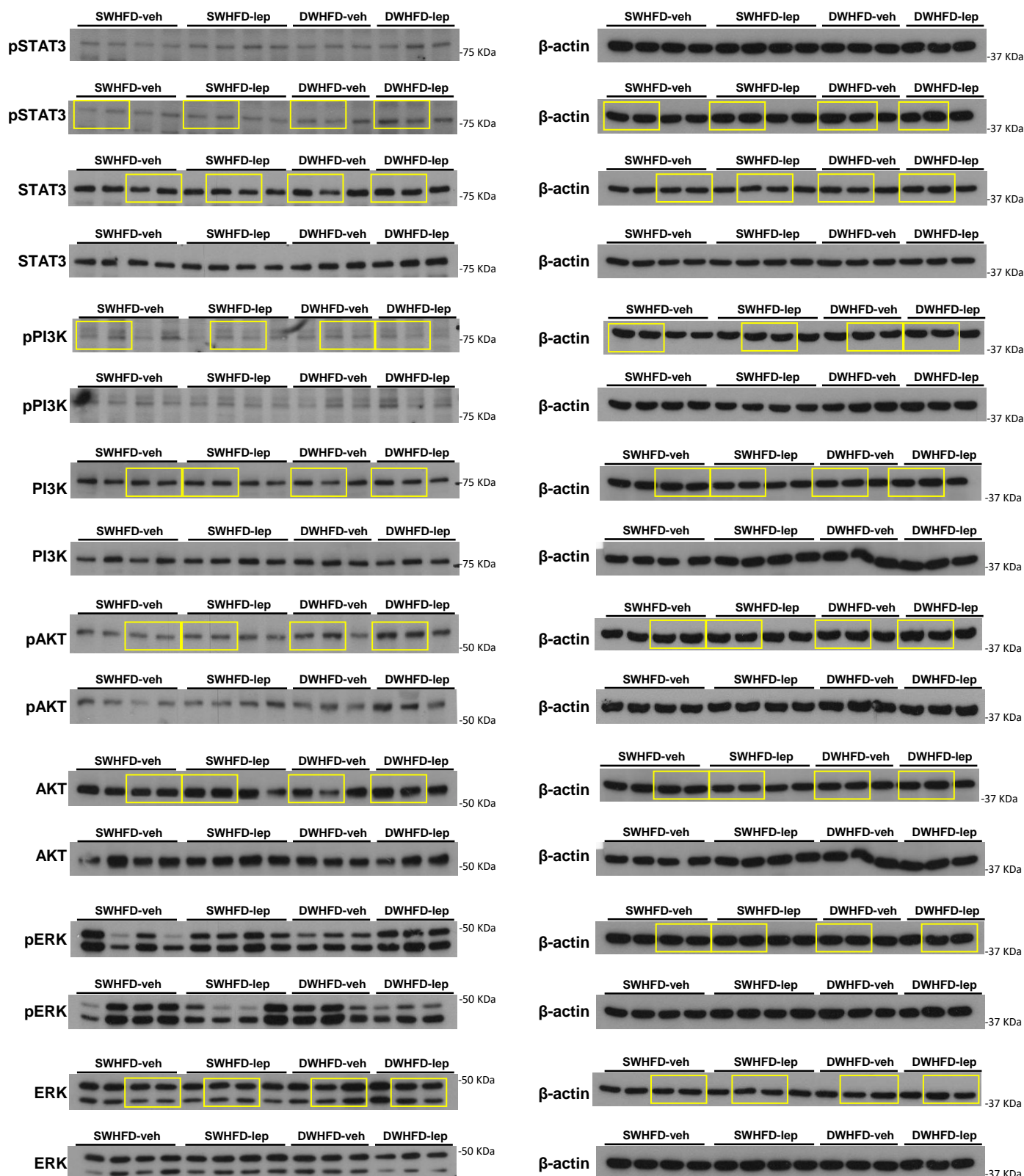

Supplement: Source Data Fig. 1 — Unprocessed western blots. [file 42255_2022_602_MOESM4_ESM.pdf]

# Supplementary Figure 2

## Uncropped blots Figure 3e

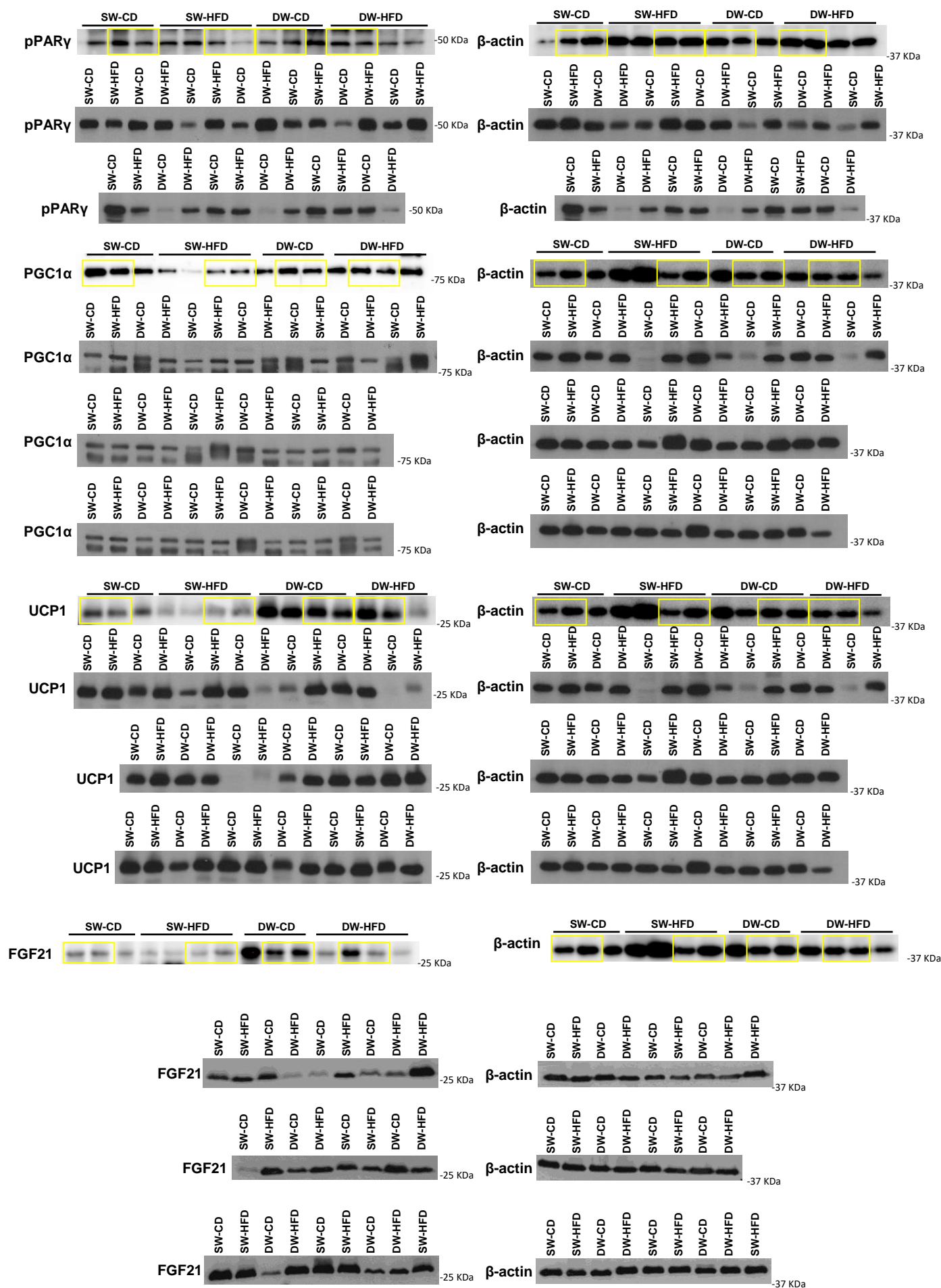

### Uncropped blots Figure 3f

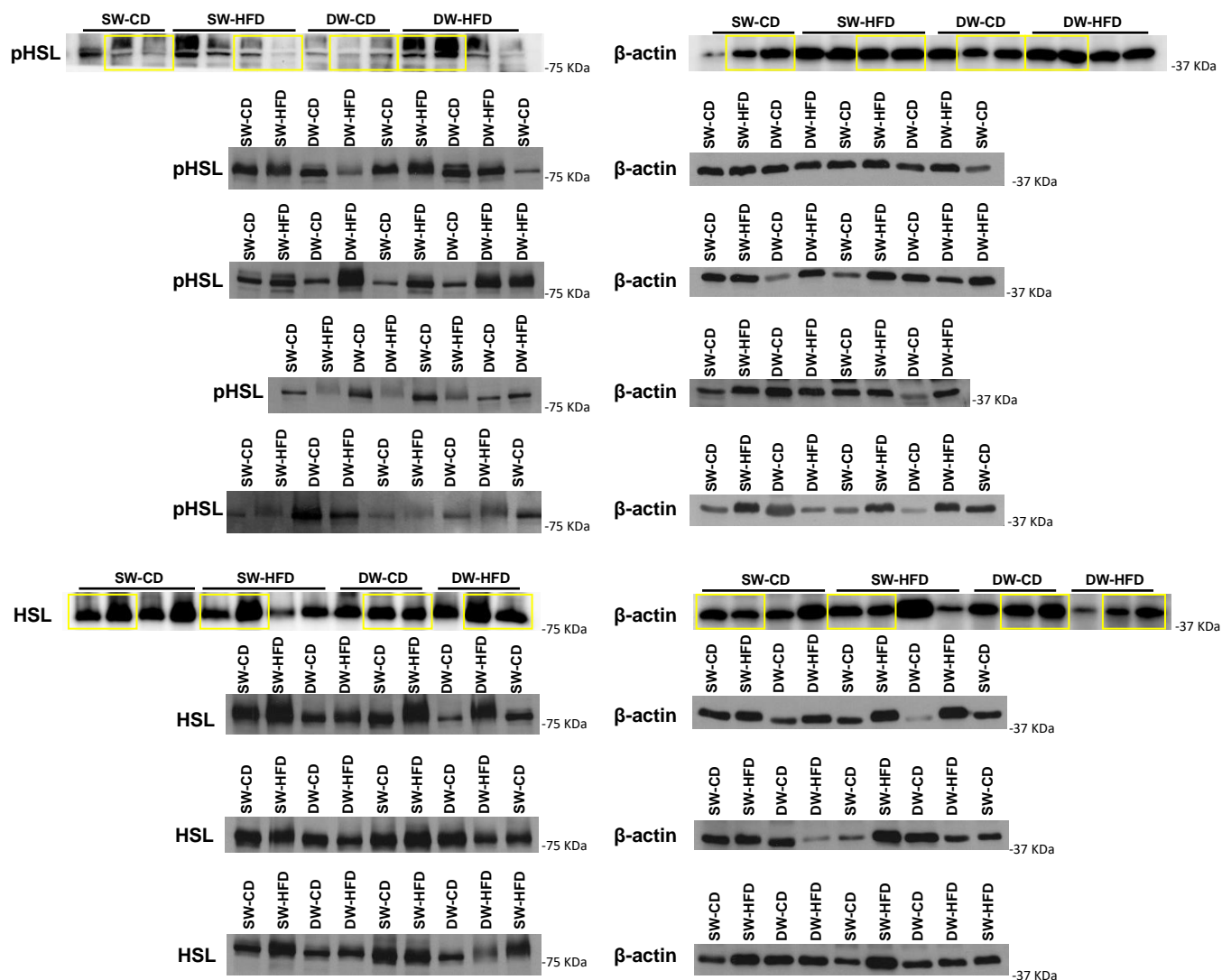

Supplement: Source Data Fig. 3 — Unprocessed western blots. [file 42255_2022_602_MOESM7_ESM.pdf]

Supplementary Figure 5

Uncropped blots Figure 6c

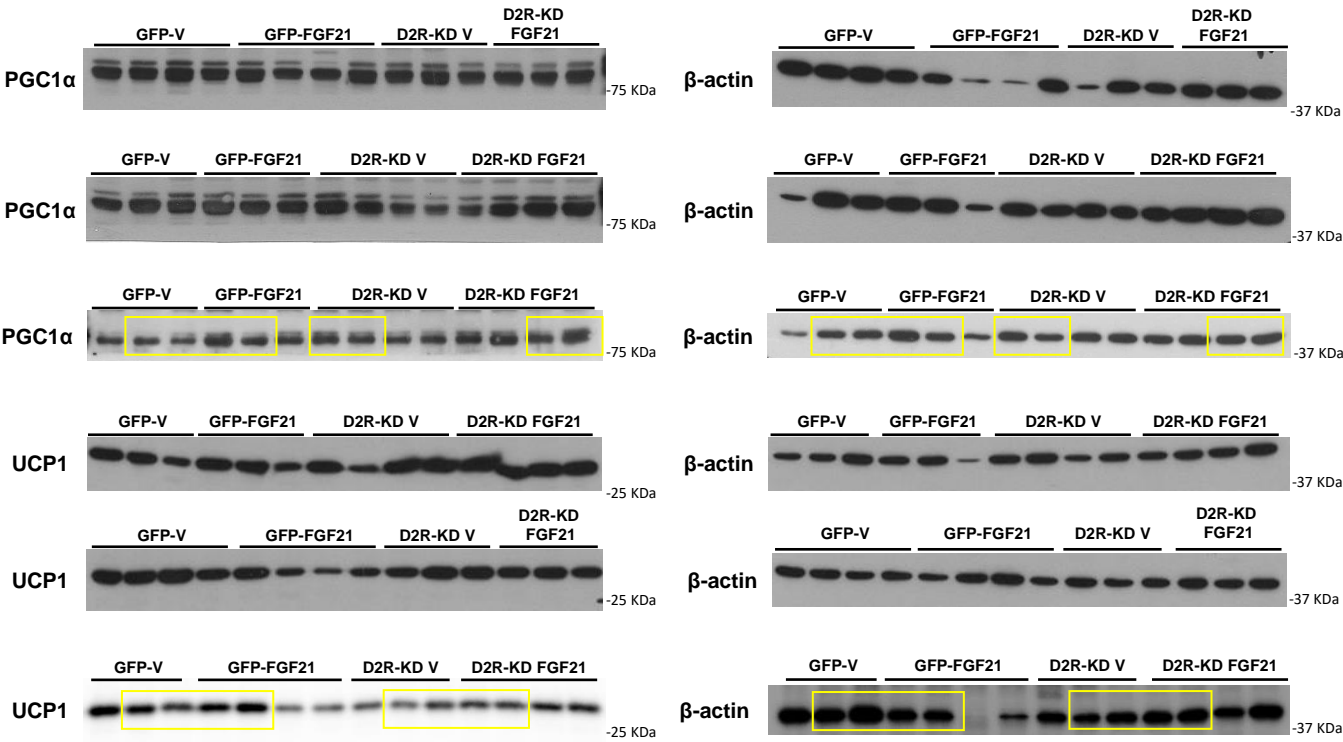

Uncropped blots Figure 6j

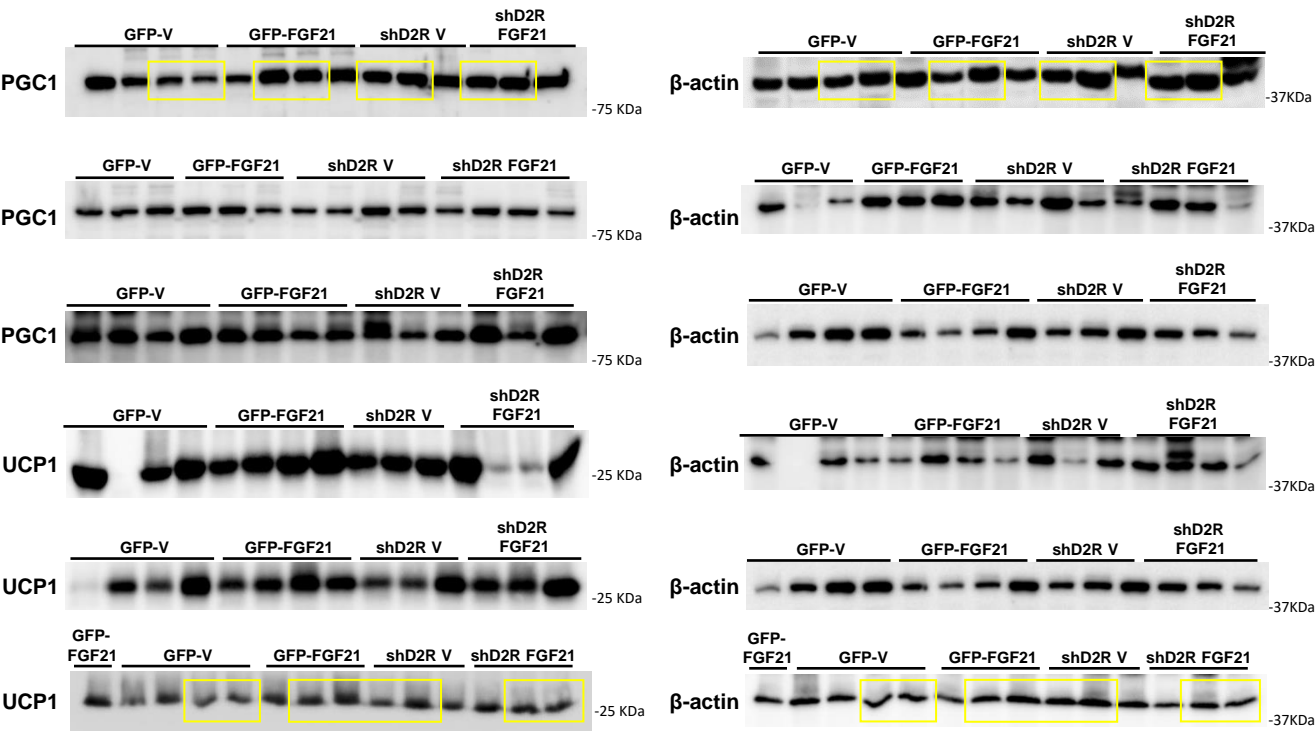

Supplement: Source Data Fig. 6 — Unprocessed western blots. [file 42255_2022_602_MOESM13_ESM.pdf]

Supplementary Figure 6

Uncropped blots Figure 7e

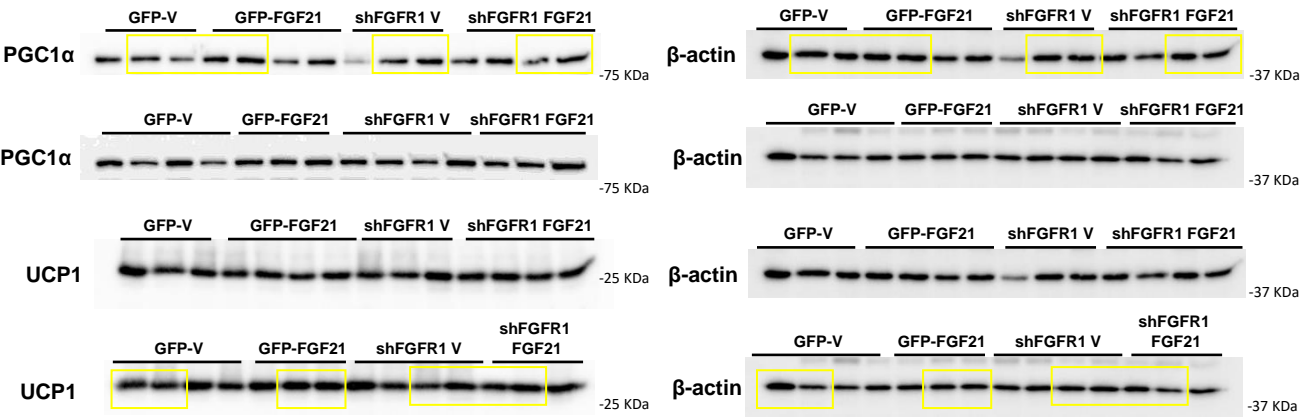

Uncropped blots Figure 7j

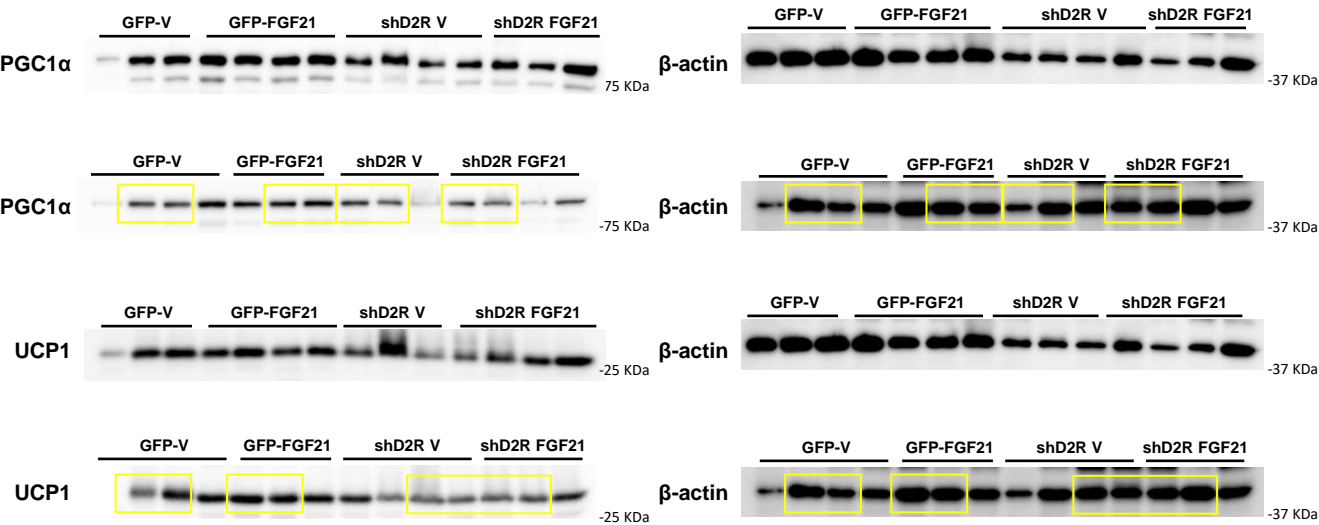

Supplement: Source Data Fig. 7 — Unprocessed western blots. [file 42255_2022_602_MOESM15_ESM.pdf]

Supplementary Figure 7

Uncropped blots Figure 8f

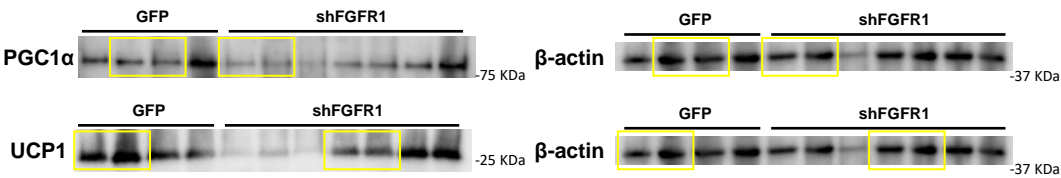

Supplement: Source Data Fig. 8 — Unprocessed western blots. [file 42255_2022_602_MOESM17_ESM.pdf]

Supplementary Figure 9

Uncropped blots Extended Data Figure 4b

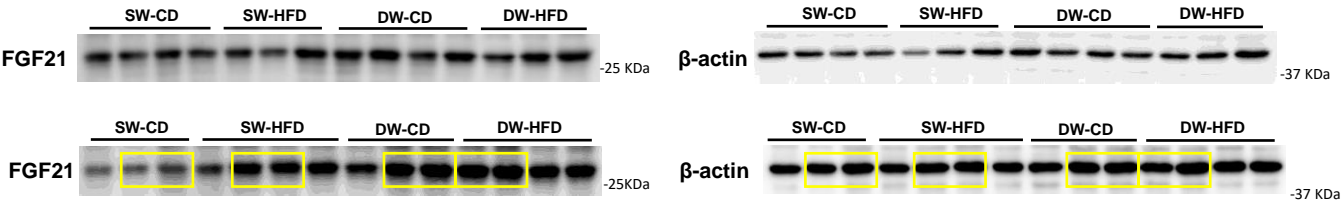

Supplement: Source Data Extended Data Fig. 4 — Unprocessed western blots. [file 42255_2022_602_MOESM23_ESM.pdf]
