# Supplementary material for: Prolonged breastfeeding protects from obesity by hypothalamic action of hepatic FGF21
Source: Nat Metab. 2022 Jul 25;4(7):901–17. doi: 10.1038/s42255-022-00602-z (PMC9314260; doi:10.1038/s42255-022-00602-z)

Supplementary Figure 10

Uncropped blots Extended Data Figure 5i

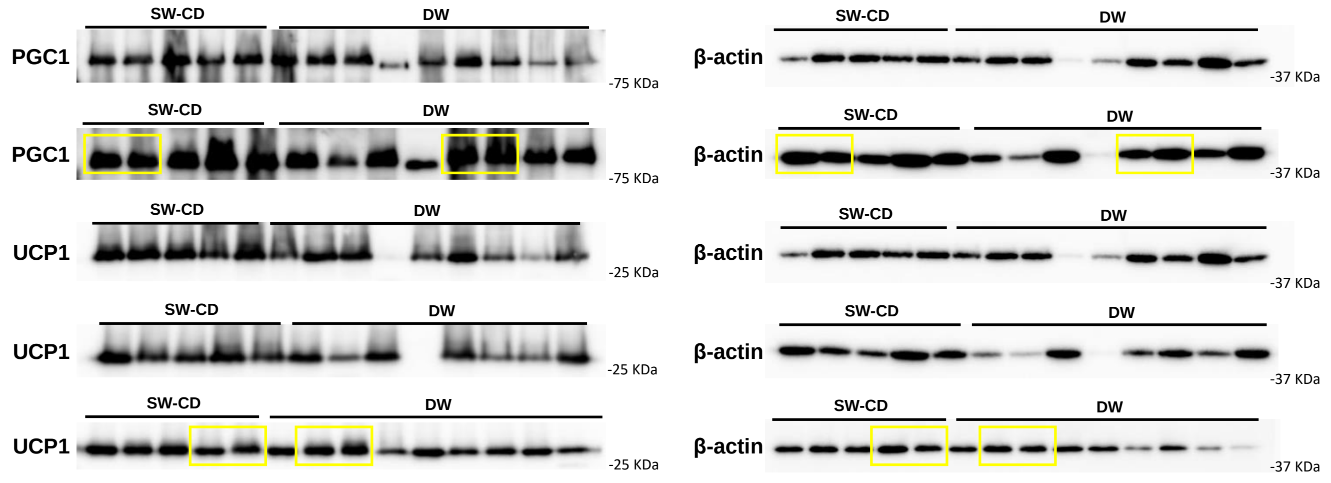

Uncropped blots Extended Data Figure 5n

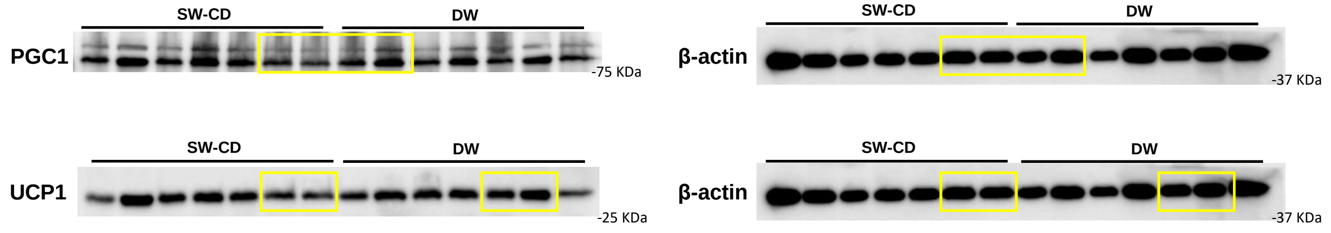

Supplement: Source Data Extended Data Fig. 5 — Unprocessed western blots. [file 42255_2022_602_MOESM25_ESM.pdf]
